# Regulation of Interface Ion Transport by Electron Ionic Conductor Construction toward High‐Voltage and High‐Rate LiNi_0.5_Co_0.2_Mn_0.3_O_2_ Cathodes in Lithium Ion Battery

**DOI:** 10.1002/advs.202402380

**Published:** 2024-06-05

**Authors:** Yunan Tian, Yuyu Li, Huasen Shen, Xiangxin Cheng, Yiming Cheng, Wen Zhang, Peng Yu, Zehui Yang, Lixing Xue, Yameng Fan, Lingfei Zhao, Jian Peng, Jiazhao Wang, Zhaohuai Li, Ming Xie, Huakun Liu, Shixue Dou

**Affiliations:** ^1^ State Key Laboratory of Precision Blasting Jianghan University Wuhan 430056 P. R. China; ^2^ Hubei Provincial Engineering Research Center of Surface and Interface Regulation Technology and Equipment for Renewable Energy Materials Jianghan University Wuhan 430056 P. R. China; ^3^ State Key Laboratory of Material Processing and Die & Mold Technology School of Materials Science and Engineering Huazhong University of Science and Technology Wuhan 430074 P. R. China; ^4^ Sustainable Energy Laboratory Faculty of Materials Science and Chemistry China University of Geosciences Wuhan Wuhan 430074 P. R. China; ^5^ Cornex New Energy Co., Ltd Wuhan 432099 P. R. China; ^6^ Institute for Superconducting and Electronic Materials Australian Institute for Innovative Materials University of Wollongong's Innovation Campus Squires Way North Wollongong NSW 2522 Australia; ^7^ Department of Mechanical and Materials Engineering Western University London ON N6A 5B9 Canada; ^8^ Institute of Energy Materials Science University of Shanghai for Science and Technology Shanghai 200093 P. R. China

**Keywords:** atomic layer deposition, electron‐ion conductor, high power density, high voltage, LiNi_0.5_Co_0.2_Mn_0.3_O_2_

## Abstract

Simultaneously achieving high‐energy‐density and high‐power‐density is a crucial yet challenging objective in the pursuit of commercialized power batteries. In this study, atomic layer deposition (ALD) is employed combined with a coordinated thermal treatment strategy to construct a densely packed, electron‐ion dual conductor (EIC) protective coating on the surface of commercial LiNi_0.5_Co_0.2_Mn_0.3_O_2_ (NCM523) cathode material, further enhanced by gradient Al doping (Al@EIC‐NCM523). The ultra‐thin EIC effectively suppresses side reactions, thereby enhancing the stability of the cathode‐electrolyte interphase (CEI) at high‐voltages. The EIC's dual conduction capability provides a potent driving force for Li^+^ transport at the interface, promoting the formation of rapid ion deintercalation pathways within the Al@EIC‐NCM523 bulk phase. Moreover, the strategic gradient doping of Al serves to anchor the atomic spacing of Ni and O within the structure of Al@EIC‐NCM523, curbing irreversible phase transitions at high‐voltages and preserving the integrity of its layered structure. Remarkably, Al@EIC‐NCM523 displays an unprecedented rate capability (114.7 mAh g^−1^ at 20 C), and a sustained cycling performance (capacity retention of 74.72% after 800 cycles at 10 C) at 4.6 V. These findings demonstrate that the proposed EIC and doping strategy holds a significant promise for developing high‐energy‐density and high‐power‐density lithium‐ion batteries (LIBs).

## Graphical abstract

In this work, ultra‐thin electron‐ion dual conductor coating on the surface and Al gradient doping in the bulk phase of NCM523 is successfully achieved by ALD, which simultaneously enhances the conductivity and stability of NCM523 at high‐voltage. This modification has enabled NCM523 to achieve high‐capacity performance and long cycle life under ultra‐high current density (1900 mAh g^−1^, 10 C) at 4.6 V.

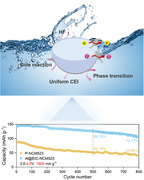

## 1 Introduction

In the era of rapid advancements in power batteries and the surging popularity of electric vehicles, the fast‐charging capability of batteries has emerged as a paramount factor influencing consumer perception and satisfaction. However, the expeditious extraction of Li^+^ presents a precarious prospect, potentially inducing structural transformations within layered cathode materials.^[^ ^1^, ^2^, ^3^ ^]^ This transformation amplifies the impedance and voltage polarization at the cathode‐electrolyte interfaces.^[^ ^4^, ^5^, ^6^ ^]^ Consequently, these challenges impede Li^+^ insertion, leading to a substantial reduction in capacity during high‐rate charging and discharging, thus hindering the pursuit of high‐power and energy density.^[^ ^7^, ^8^, ^9^ ^]^ Furthermore, the utilization of commercial nickel‐enriched layered cathode materials, such as Li[Ni_x_Co_y_Mn_1‐x‐y_]O_2_ (NCM), is hindered by inferior conductivity,^[^ ^10^, ^11^, ^12^ ^]^ further obstructs the migration of Li^+^ ions at the interface, and impedes the intricate dynamics of the involved reactions. As a result, high‐power Lithium‐ion batteries (LIBs) are endowed with an expanded array of challenges to surmount.

Recognizing that the cost of batteries constitutes nearly half of the total vehicle price in electric vehicles, with the cathode materials alone accounting for 40% of the overall raw materials costs, it becomes imperative to consider not only the electrochemical performance of electrode materials but also their economic implications. Li[Ni_0.5_Co_0.2_Mn_0.3_]O_2_ (denoted as NCM523), as a ternary cathode material, distinguishes itself for superior safety, structural stability, and cost‐effectiveness.^[^ ^13^, ^14^, ^15^ ^]^ Furthermore, the capacity of NCM523 can be elevated to 200 mAh g^−1^ when the cut‐off voltage is pushed to a remarkable 4.6 V,^[^ ^16^, ^17^, ^18^ ^]^ comparable to that of Li[Ni_0.8_Co_0.1_Mn_0.1_]O_2_(denoted as NCM811) (4.2 V) with a higher nickel content,^[^ ^19^, ^20^, ^21^ ^]^ while maintaining superior thermal stability and cost‐effectiveness. Unfortunately, the ternary layered oxide cathode materials undergo irreversible H2‐H3 phase transitions at elevated voltages (>4.3 V),^[^ ^22^, ^23^, ^24^ ^]^ resulting in the profound disintegration of the layered structure and unfavorable Li/Ni mixing.^[^ ^25^, ^26^, ^27^ ^]^ Additionally, these conditions promote oxygen release from the crystal lattice, thereby intensifying the interface reactions with the electrolyte and precipitating an abrupt escalation of interface impedance polarization effects,^[^ ^28^, ^29^, ^30^ ^]^ thereby restricting the rate capability and optimal utilization capacity of NCM523. In order to effectively suppress the crystal structure degradation and interface instability of nickel‐rich layered oxide cathode, Jiang and co‐workers^[^ ^31^ ^]^ employed oxalate‐assisted deposition strategy to fabricate a LiAlO_2_ coating layer on its surface, as well as incorporating Al doping in the bulk phase, resulting in an enhanced cycling stability. In a more recent study conducted by Zheng and co‐workers^[^ ^32^ ^]^ the Li/Ni mixing effect in NCM523 cathode material was successfully mitigated using groundbreaking Li^+^ and Na^+^ ion exchange method. However, the optimization of performance and the comprehensive elucidation of underlying mechanisms in the NCM523 material, which exhibits immense promise for commercial applications, continue to present a dearth of progress particularly under high‐voltage (≥4.5 V) and high‐rate (≥ 10 C) conditions. Furthermore, it is crucial that the methods employed for material modification possess practical scalability, thus ensuring widespread applicability.

In this work, we employed scalable atomic layer deposition (ALD) technology (kilogram‐scale) to construct an ultra‐thin (0.6 nm) protective coating of electronic and ionic dual continuous conductor (EIC) on the surface, combined with synergistic Al doping in the bulk phase of commercial NCM523 (Al@EIC‐NCM523). The aluminum phosphate (EIC: AlPO_4_) protecting layer, obtained by ALD deposition and phase transformation treatments, demonstrates exceptional compactness and electrochemical stability. It plays a pivotal role in mitigating detrimental cathode‐electrolyte interactions, thereby enhancing the cathode‐electrolyte interphase (CEI) and ensuring anodic stability under high‐voltage conditions. In addition, the EIC outperforms pristine NCM523 (P‐NCM523) with superior electronic conductivity and a lower diffusion barrier for Li^+^ ions. The integration of AlPO_4_ EIC achieved through a solid‐state heat treatment process, promotes seamless interface compatibility and introduces an Al gradient doping, facilitating Li^+^ ions diffusion and expediting ion de‐intercalation pathways in NCM523. This innovative route significantly enhances the structural integrity and suppresses phase transitions in Al@EIC‐NCM523 at critical high‐voltages. Remarkably, Al@EIC‐NCM523 demonstrates 62% improvement in reversible capacity (114.7 mAh g^−1^) at an ultra‐high rate of 20 C (1.9 A g^−1^) and 4.6 V. Moreover, in practical application, the full pouch cell using Al@EIC‐NCM523 as cathode materials demonstrate an exceptional balance of high‐power and energy density, maintaining ultra stable cycling life up to nearly 1000 cycles. These pioneering findings not only highlight the transformative potential of Al@EIC‐NCM523 but also set a new benchmark for high‐power and high‐voltage LIBs.

## 2 Results and Discussion

**Figure 1:**
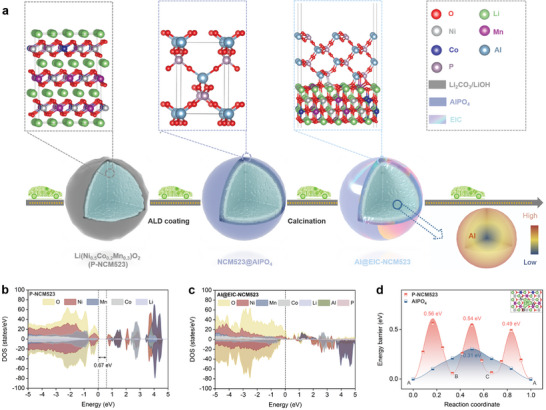
Schematic of preparing process and theoretical calculations. a) Schematic of the synthesizing method of Al@EIC‐NCM523. DOS values of selected elements and total elements of b) P‐NCM523 and c) Al@EIC‐NCM523. d) Diffusion energy barriers of Li^+^ ions in P‐NCM523 and AlPO_4_ along different paths.

To achieve NCM523 cathode material that exhibits both high‐voltage stability and high‐rate performance, this work initially employs the precise encapsulation capability of ALD technique to construct a P‐ and Al‐ compound precursor coating on the surface of the P‐NCM523.^[^ ^33^ ^]^ Subsequently, a solid‐phase heat treatment is performed in the air atmosphere, combining thermal molecular diffusion and oxidation processes, resulting in an Al penetrated effect and AlPO_4_ EIC surface coating with a thickness of only 0.6 nm, which exhibits high electronic conductivity, low ionic diffusion barrier, and a smooth transition at the solid‐phase interface between EIC coating layer and NCM523 surface (**Figure** **1**a). Theoretical calculations and molecular structure simulations demonstrate a robust chemical bonding between AlPO_4_ and NCM523 (Figure 1a), indicating a structurally compatible interface between the EIC layer and NCM523. The NCM523 ($\bar{1}$01) and AlPO_4_ (100) lattice planes were matched to construct the NCM523/AlPO_4_ interface structure. Four layers of atoms were used for each phase (Figure S1, Supporting Information). This well‐designed EIC layer could effectively suppress side reactions between the electrolyte and the cathode material, as well as mitigate stress concentration caused by rapid Li^+^ insertion/extraction. Furthermore, Density of States (DOS) calculations reveal that the P‐NCM523 possesses a noticeable bandgap of 0.67 eV (Figure 1b), in agreement with prior findings.^[^ ^34^, ^35^ ^]^ However, following the Al gradient doping and EIC encapsulation, the valence band of Al@EIC‐NCM523 approaches the Fermi level. This proximity results in the elimination of the bandgap, endowing the material with metallic characteristics and a zero bandgap (Figure 1c). Consequently, there is a notable enhancement in the electronic conductivity of Al@EIC‐NCM523. On the other hand, due to the intrinsic low ionic conductivity of NCM‐based materials and the presence of residual alkaline species on their surface, the migration barrier and impedance polarization effects for Li^+^ ions are substantial, hindering the rapid insertion/extraction of Li^+^.^[^ ^36^ ^]^ To investigate the Li^+^ transfer ability along the target materials, the calculated Li^+^ ions migration barriers in P‐NCM523 and Al@EIC‐NCM523 bulk phases (Figure S2, Supporting Information) show that the migration barrier in EIC (0.31 eV) is lower than that in P‐NCM523 (0.56 eV), implying a more rapid Li diffusion within the EIC coating (Figure 1d). We assume that the Al@EIC‐NCM523 has great potential in accelerating rapid transport of Li^+^ across the Al@EIC‐NCM523 interface, establishing an efficient pathway for ion transportation within the NCM523 bulk phase and achieving high‐efficiency Li^+^ insertion/extraction capabilities toward high‐power LIBs.

**Figure 2:**
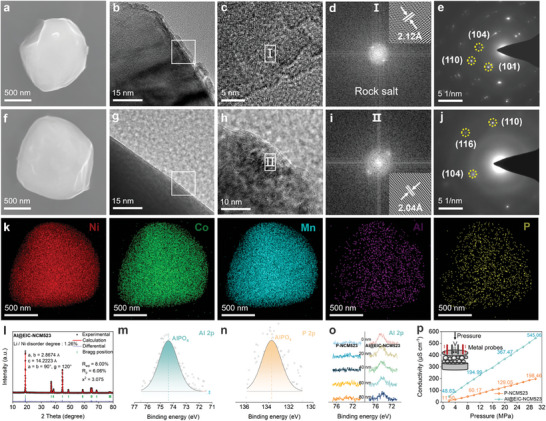
Structural properties and characterizations. SEM images of a) P‐NCM523 and f) Al@EIC‐NCM523. TEM images at the near‐surface area and corresponding FFT patterns of b–e) P‐NCM523 and g–j) Al@EIC‐NCM523 particles. k) Corresponding EDS mapping of Al@EIC‐NCM523. l) Rietveld refinement of the XRD patterns for Al@EIC‐NCM523. m) Al 2p and n) P 2p XPS spectra of Al@EIC‐NCM523. o) XPS spectra of Al 2p with different depths in P‐NCM523 and Al@EIC‐NCM523. p) The electric conductivity of P‐NCM523 and Al@EIC‐NCM523 powders under different pressures measured by four‐probes method.

To explore the structure and morphology of as‐prepared Al@EIC‐NCM523, scanning electron microscopy (SEM) analysis reveals no discernible differences in the microstructural morphology between P‐NCM523 and Al@EIC‐NCM523 (**Figure** **2**a,f), indicating that the thermally treated NCM523 maintains the integrity of its single crystal structure. While the particle size distribution shows that in the captured image, the single crystal particles are tightly arranged, the Al@EIC‐NCM523 particles are slightly larger than those of P‐NCM523, the average particle size of P‐NCM523 is 3.58 µm, the Al@EIC‐NCM523 particles are more uniformly distributed with an average particle size of 4.17 µm (Figure S3, Supporting Information). Transmission electron microscopy (TEM) analysis provides a comprehensive investigation into P‐NCM523 and Al@EIC‐NCM523, revealing conspicuous phase separation on the surface of P‐NCM523 (Figure 2b,c). Correspondingly, selected‐area electron diffraction (SAED) results indicate the presence of a rock salt phase on the P‐NCM523 surface with a characteristic crystal plane orientation of (002) (Figure 2d,e), hinting at the existence of a substantial number of residual alkali species.^[^ ^37^ ^]^ This observation suggests the potential existence of a large interfacial impedance and unfavorable kinetics of Li^+^ interface reactions in electrochemical processes. Conversely, the surface of NCM523 coated with the EIC exhibits a smooth and undifferentiated interface, signifying the perfect compatibility between the EIC and the NCM523 surface structure (Figure 2g,h). Moreover, the SAED result confirms the presence of a well‐defined (104) crystal plane in Al@EIC‐NCM523 (Figure 2i,j),^[^ ^38^ ^]^ ensuring the electrochemical activity of the Al@EIC‐NCM523 interface and promoting faster Li^+^ ions migration. The elemental distribution mappings demonstrate a concentrated presence of P primarily in the outer EIC layer, while Al permeates throughout the entire NCM523 bulk, providing additional evidence for the successful preparation and acquisition of Al@EIC‐NCM523 (Figure 2k; Figure S4, Supporting Information).

The crystal structures information of the P‐NCM523 and Al@EIC‐NCM523 are investigated by X‐ray Diffraction (XRD) patterns, and both materials are free of impurity phases and correspond to the hexagonal α‐NaFeO_2_ structure of space group R‐3m (Figure 2l; Figure S5, Supporting Information).^[^ ^39^ ^]^ The pairs of peaks (006), (102) and (108), (110) of both the P‐NCM523 and Al@EIC‐NCM523 are well separated and show a highly ordered layered structure. The crystallographic parameters and atomic occupancies of P‐NCM523 and Al@EIC‐NCM523 are then obtained by XRD Rietveld refinement (Table S1, Supporting Information), which exhibits the lattice parameters (a = 2.8674 Å and c = 14.2223 Å) of Al@EIC‐NCM523 are slightly reduced compared with the P‐NCM523 (a = 2.8677 Å and c = 14.2232 Å), confirming that Al element is successfully doped in the crystalline lattice of Al@EIC‐NCM523 after ALD deposition and thermal treatment processes. Remarkably, the meticulously refined XRD spectra unveil a remarkable finding: the proportion of Ni in the Li layer in P‐NCM523 amounts to 4.42% of the composition, contrasting with a mere 1.26% in Al@EIC‐NCM523. This compelling evidence indirectly verifies that the incorporation of Al dopants in the bulk phase confers stability upon the arrangement of Li and Ni atoms, as well as the layered structure of Al@EIC‐NCM523. As a result, this augmentation substantially enhances the structural resilience of NCM523 cathode materials when subjected to high‐voltage conditions, effectively safeguarding the expeditious de‐lithiation of its layered architecture.

To delve deeper into the elemental information of the EIC coating, X‐ray photoelectron spectroscopy (XPS) analysis proves the successful construction and encapsulation of AlPO_4_ EIC layer (Figure 2m,n; Figure S6a, Supporting Information). The fitting results of Li 1s spectra reveal an abundance of LiOH and Li_2_CO_3_ species on the surface of P‐NCM523 compared to Al@EIC‐NCM523 (Figure S6b, Supporting Information).^[^ ^40^ ^]^ This compelling evidence solidifies the notion that the EIC protective layer not only expedites the diffusion of Li^+^ ions but also enhances the air stability of NCM523, which is consistent with the TEM results (Figure 2b,g). In addition, the Ni 2p spectra demonstrate a lower proportion of Ni^2+^ components, further corroborating the mild occurrence of Li/Ni mixing on the surface of Al@EIC‐NCM523 (Figure S6c, Supporting Information). This favorable phenomenon is conducive to the reduction of migration barriers and impedance encountered by Li^+^ ions at the interface. Furthermore, the Al 2p XPS spectra obtained through argon ion etching reveal that the binding energy of Al remains stable as the etching depth increases (Figure 2o). This observation provides strong evidence of the distribution of Al in the bulk phase of NCM523, which constitutes the fundamental mechanism behind the preservation of NCM523's crystal structure integrity and the suppression of Li/Ni mixing. The conductivity measurements of P‐NCM523 and Al@EIC‐NCM523 are performed under different pressures in the interval of 2–30 MPa (Tables S2 and S3, Supporting Information) by four‐point probes. The overall electronic conductivity trended to increase linearly with pressure added due to the increase in compaction density (Figure 2p). The results of four‐point probes show that a high electronic conductivity of Al@EIC‐NCM523 up to 545.06 µS cm^−1^ at 30 MPa while the P‐NCM523 is only 198.46 µS cm^−1^, demonstrating that the electronic conductivity of the NCM523 powder is effectively enhanced by surface construction and doping strategy.

**Figure 3:**
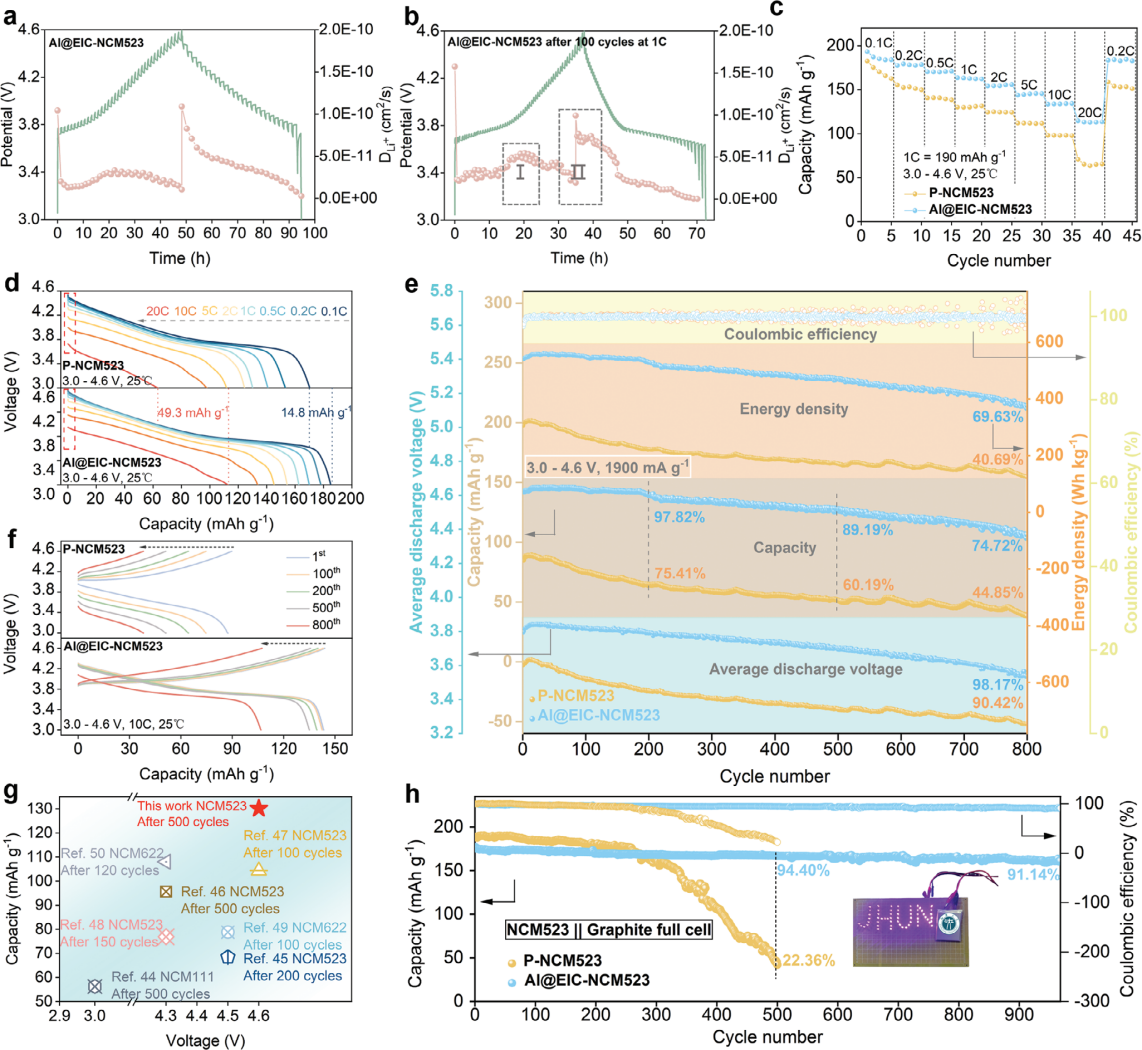
Electrochemical performances of the cathodes in half‐/full‐cells. GITT measurements of Al@EIC‐NCM523 during the a) initial and b) after 100 cycles at 1 C. c) Rate performance of the half cells with P‐NCM523 and Al@EIC‐NCM523 at a cut‐off voltage of 4.6 V and d) corresponding charge–discharge curves. e) Comparison of rapid charge and discharge performance of the NCM || Li half‐cells with P‐NCM523 and Al@EIC‐NCM523 between 3.0 and 4.6 V. and f) corresponding charge–discharge curves. g) Comparison of the cycling performance of the Al@EIC‐NCM523 at a high‐rate with NCM in previous reports.^[^ ^44^, ^45^, ^46^, ^47^, ^48^, ^49^, ^50^ ^]^ h) Comparison of cycling performance of the NCM || graphite full cells with P‐NCM523 and Al@EIC‐NCM523 at 0.5 C between 3.0 and 4.5 V.

To investigate the interface ion transport modulation mechanism of EIC in Al@EIC‐NCM523, we first employed cyclic voltammetry (CV) over the voltage range of 3.0–4.6 V to study the electrochemical kinetics of Al@EIC‐NCM523||Li half cells (Figure S7, Supporting Information). The test results reveal that at an identical scan rate of 0.1 mV s^−1^, Al@EIC‐NCM523 showcased heightened stability in charge–discharge potentials (ΔE = 0.11 V) and greater response current values (I = 1.1 mA) in contrast to P‐NCM523 (ΔE = 0.12 V, and I = 0.49 mA). These observations consistently indicate a diminished occurrence of electrochemical polarization effects alongside accelerated electrochemical kinetics within the Al@EIC‐NCM523 system. Additionally, the line‐scan voltammetry (LSV) testing has revealed the exceptional electrochemical robustness of AlPO_4_ within the voltage range of 0–5.0 V (Figure S7, Supporting Information). This remarkable stability guarantees that AlPO_4_, serving as a protective barrier, can proficiently segregate the cathode from the electrolyte, thus upholding a steady CEI during prolonged cycling. Multi‐scan‐rate CV tests, with scan rates increasing from 0.1 to 1.6 mV s^−1^, showing that Al@EIC‐NCM523 demonstrates smaller charge–discharge voltage biases compared to P‐NCM523 (Figure S9a,b, Supporting Information), further confirming the ability of EIC and P‐phase doping in Al@EIC‐NCM523 to achieve more stable interfacial Li^+^ redox reactions and suppress voltage polarization effects.^[^ ^41^ ^]^ The corresponding linear relationship between the peak current (i_p_) and the square root of the scan rate (v^1/2^) is shown in Figure S9c (Supporting Information), which also strongly supports the faster Li^+^ transport rate in Al@EIC‐NCM523, providing the driving force for high‐rate Al@EIC‐NCM523 LIBs. In order to further explore the Li^+^ intercalation and de‐intercalation behaviors of samples, galvanostatic intermittent titration technique (GITT) measurements were performed. Compared to the initial ones (**Figure** **3**a; Figure S10a, Supporting Information), the cycled P‐NCM523 and Al@EIC‐NCM523 have higher D_Li_+ values (Figure 3b; Figure S10b, Supporting Information). On the one hand, the lower D_Li_+ values during the initial charge section and discharge section, which is related to the formation of the H2 phase with the poor Li^+^ transport capacity due to the narrowed interlayer spacing.^[^ ^42^ ^]^ On the other hand, the slow migration rate of Li^+^ exacerbates the internal strain generated by the H2‐H3 transition during the final charge section, leading to structural degradation and hindering ion diffusion, resulting in inhomogeneous distribution. The use of AlPO_4_ can effectively improve the diffusion kinetics of Li^+^ at the surface and interface, which leads to a more homogeneous distribution of Li^+^, thus alleviating the internal stress, which is consistent with the previous reports. As can be seen from the figures, Al@EIC‐NCM523 has a smaller total voltage change (ΔEt) and bigger voltage evolution of the steady state for the related step (ΔEs) compared to P‐NCM523, indicating that the diffusion ability of Li^+^ is improved and the polarization is suppressed.^[^ ^43^ ^]^ Overall, Al@EIC‐NCM523 has higher ion diffusion coefficients during the charging and discharging, such as region I and region II (marked by the grey shadows), which is conducive to the rate performance.

The performance of Al@EIC‐NCM523 LIBs serves as a crucial benchmark for assessing the Al gradient doping and the EIC ion transport regulation dual effects. Initially, at a diminutive current density of 0.1 C (1 C = 190 mAh g^−1^), the initial discharge capacities of P‐NCM523 and Al@EIC‐NCM523 at a high cut‐off voltage of 4.6 V are found to be 184.2 mAh g^−1^ and 195.8 mAh g^−1^ (Figure S11, Supporting Information), respectively, which not only substantiates the capacity performance of a medium‐nickel ternary cathode material (NCM523), on par with high‐nickel (Ni ≥ 80%) NCM cathode materials under elevated voltages but also correlates with their respective charge–discharge curves, showcasing Al@EIC‐NCM523's diminished polarization voltage and a harmoniously smooth charge–discharge plateau. In addition, the rate capability and corresponding discharge profiles of Al@EIC‐NCM523 are exemplified by its ability to maintain a stable reversible capacity of 114.7 mAh g^−1^ at 4.6 V, even under the extreme high‐rate condition of 20 C (Figure 3c,d), which represents an impressive 62% increase in capacity compared to P‐NCM523, providing compelling evidence of Al@EIC‐NCM523's potential to advance high‐voltage and high‐power LIBs applications.

The long‐term cycling performance under high‐voltage and high‐rate conditions is another crucial criterion for practical battery applications. Therefore, we conducted cycling tests on P‐NCM523 and Al@EIC‐NCM523 cathodes using discharge and charge current densities of 5 C and 10 C respectively. It's obvious that the cycling performance of Al@EIC‐NCM523 (82.81% after 400 cycles) is much better than that of P‐NCM523 (31.57% after 400 cycles) at 5 C (Figure S12, Supporting Information), and it further exemplifies the superior cycling performance and advantageous energy density at a significantly elevated current density of 10 C (Figure 3e,f). Substantially, in comparison to the electrochemical characteristics of Ni‐based ternary layered cathode materials reported previously at high cut‐off voltages (≥4.5 V), the Al@EIC‐NCM523 exhibits exceptional cycling stability even at an elevated cut‐off voltage of 4.6 V and under current densities reaching 10 C (Figure S13 and Table S4, Supporting Information). To our current understanding, the Al@EIC‐NCM523 demonstrates unparalleled rate performance and exceptional cycling durability, surpassing all other known ternary layered materials in these aspects (Figure 3g; Table S5, Supporting Information).^[^ ^44^, ^45^, ^46^, ^47^, ^48^, ^49^, ^50^ ^]^ The exceptional combination of high‐voltage, high‐rate capability, and long cycling lifespan achieved by Al@EIC‐NCM523 can be attributed to three factors: 1) The enhanced Li^+^ ions diffusion ability of EIC induces the establishment of a rapid Li^+^ insertion/extraction pathway within the NCM523 bulk phase. 2) The dense and electrochemically stable EIC effectively inhibits surface rock‐salt transformation and stabilizes the CEI. 3) The gradient doping of P enhances the atomic arrangements of Li/Ni, suppressing irreversible phase transitions of NCM under high‐voltage conditions and synergistically enabling a LIB with high‐voltage stability and fast ion reaction kinetics through the collaboration with EIC.

To further substantiate the practical application of our proposed Al@EIC‐NCM523 in LIBs, this study employed P‐NCM523 and Al@EIC‐NCM523 as the cathode materials, and commercial graphite as the anode material to fabricate full pouch cells with a capacity of 150 mAh (Figure 3h). To more authentically reflect the prospects of Al@EIC‐NCM523's application in LIBs, the areal loading of the cathodes (P‐NCM523 and Al@EIC‐NCM523) in pouch cells reaches a remarkable value of 3.17 mAh cm^−2^, the electrolyte/capacity (E/C) ratio is 3.00 g Ah^−1^, the negative electrode capacity/positive electrode capacity (N/P) ratio is designed to be 1.17 and the testing voltage range spans from 3.0 to 4.5 V (Table S6, Supporting Information). As a result, the P‐NCM523||graphite pouch cell shows rapid capacity fading with extremely low capacity retention of 22.36% after 500 cycles at 0.5 C, while the capacity retention of Al@EIC‐NCM523||graphite pouch cell still remains 91.14% after 968 cycles, demonstrating practical requirements close to 1000 cycles of commercial battery life with capacity retention above 80%. This further confirms that the gradient doping of Al and the induced interface‐controlled ion transfer by EIC significantly enhance the structural stability of the NCM523 electrode material and the cycling lifespan of high‐voltage LIBs.

**Figure 4:**
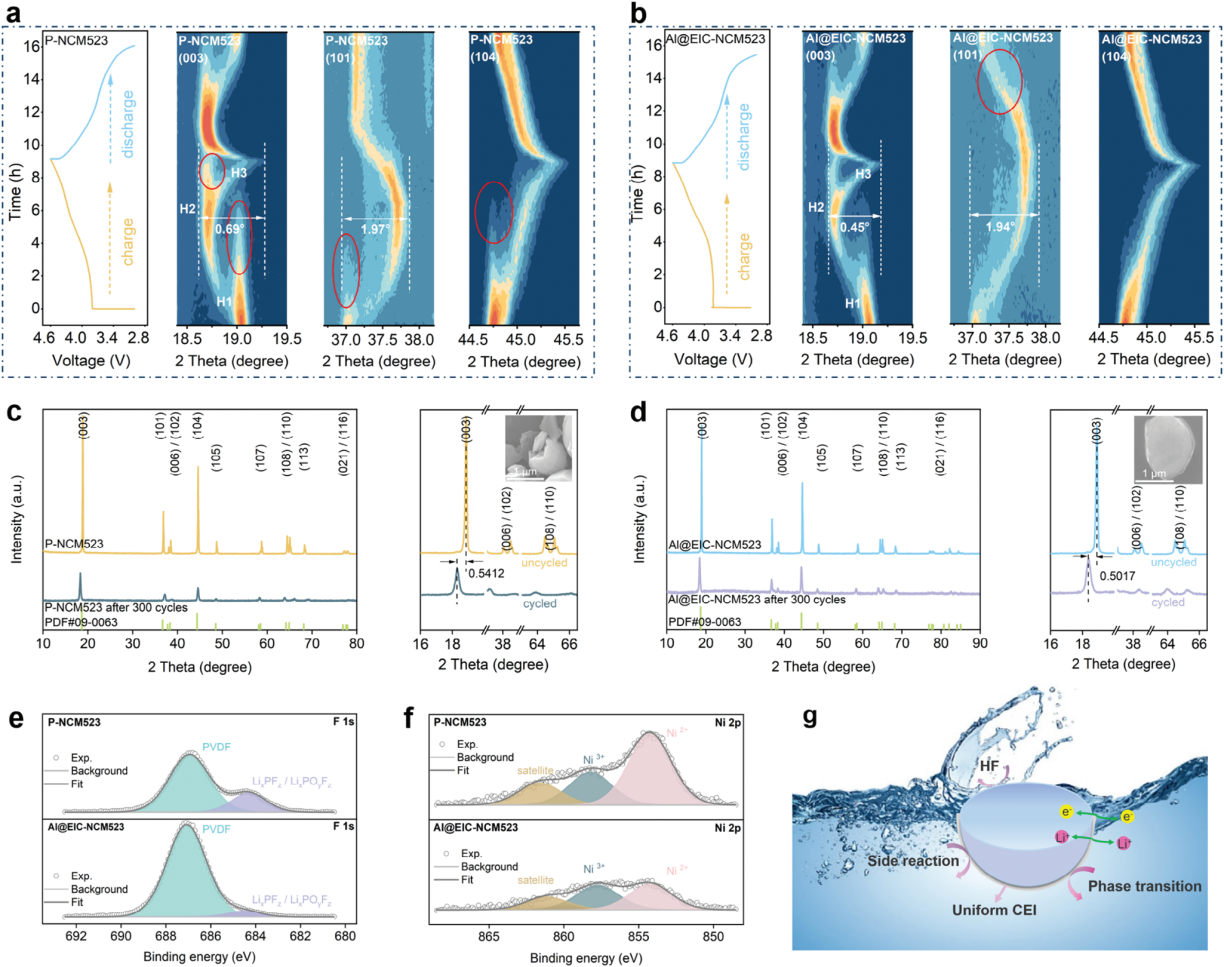
Structural stability characterization and degradation mechanism. In situ XRD patterns and corresponding charge–discharge curves of a) P‐NCM523 and b) Al@EIC‐NCM523 during the first charge and discharge process at 0.1 C between 3.0 and 4.6 V. XRD patterns of c) P‐NCM523 and d) Al@EIC‐NCM523 electrodes before and after 300 cycles at 10 C and 25 C. XPS spectra of e) F 1s and f) Ni 2p for P‐NCM523 and Al@EIC‐NCM523 cathodes after 300 cycles at 10 C and 25 C. g) Schematic of degradation mechanism of Al@EIC‐NCM523.

In order to further investigate the effect of structure evolution and phase transformation that derive from EIC construction on the electrochemical performance, this study employs in situ XRD measurements of P‐NCM523 and Al@EIC‐NCM523 half cells, operating at a cut‐off voltage of 4.6 V and a current density of 0.1 C (Figure S14, Supporting Information). The Al@EIC‐NCM523 exhibits an offset maximum of 0.45° for (003) reflection during phase transition from H2 to H3, which is smaller than that of P‐NCM523 (0.69°) (**Figure** **4**a,b), indicating that lattice contraction of the c‐axis is suppressed after EIC surface reconstruction and Al gradient doping. Meanwhile, the obviously reduced offsets of (101) and (104) peaks are also observed for Al@EIC‐NCM523 over P‐NCM523, while the variation of c and a axes of Al@EIC‐NCM523 are 4.7% and 1.8%, respectively, which are all smaller than that of P‐NCM523, illustrating that Al@EIC‐NCM523 is beneficial to the mitigation of a‐axis and c‐axis contraction during fast Li^+^ inserting and extracting. Notably, the in situ XRD patterns demonstrate a more prominent bulge in contour maps of P‐NCM523 compared with that of Al@EIC‐NCM523, which suggests that the kinetics for the H1‐H2 phase transition of P‐NCM523 is more sluggish than that of Al@EIC‐NCM523 (Figure S15, Supporting Information). It is believed that the H1‐H2 phase transition is caused by the changes in Li content in the lattice of Ni‐based ternary layered cathode materials during charge process. Consequently, the kinetics for the H1‐H2 phase transition is determined by the rate of de‐lithiation in charge process of NCM523, which is related to Li^+^ ions diffusion kinetics in NCM523 cathode materials.^[^ ^51^ ^]^ Therefore, the coexistence of H1 and H2 phases in P‐NCM523, compared to the sole presence of the H2 phase in Al@EIC‐NCM523 when charged to 3.7 V, suggests that the Li^+^ ions diffusion kinetics during the charging process are more sluggish in P‐NCM523 than in Al@EIC‐NCM523. Furthermore, a similar phenomenon occurred in other potential versus Li^+^ in charge and discharge processes of P‐NCM523 and Al@EIC‐NCM523 (areas circled in red, in Figure 4a), indicates that Li^+^ ions diffusion kinetics in charge and discharge process of NCM523 are efficiently improved by Al@EIC design, which is coincided with the results of CV test (Figure S9, Supporting Information).

Furthermore, the volumetric changes in the crystal lattices of both P‐NCM523 and Al@EIC‐NCM523 indicate that after one cycle, P‐NCM523 experiences a maximum volume change of 9.6% relative to its initial volume. In contrast, Al@EIC‐NCM523 exhibits a volume change of 9.1% (Figure S16, Supporting Information). This observation directly demonstrates the effective restriction of volume and structure variations achieved through EIC and Al doping optimizations during the high‐rate cycling process. In addition, from the amplified (003) signal of P‐NCM523 and Al@EIC‐NCM523 before and after 300 cycles at 10 C rate, the peak location offset of Al@EIC‐NCM523 is 0.50° after 300 cycles, slightly less than that of P‐NCM523 (0.54°), illustrating that the higher layer structure maintenance in Al@EIC‐NCM523 compared to P‐NCM523. Correspondingly, the SEM images of P‐NCM523 and Al@EIC‐NCM523 suggest that the P‐NCM523 displays obvious cracks and coarse surface after 300 cycles but Al@EIC‐NCM523 after 300 cycles exhibited a smoother surface with no obvious crack on the surface, which is caused by notorious side reaction on electrode/electrolyte interface and anisotropic lattice volumetric variation of P‐NCM523 (Figure 4c,d). The XPS results of cycled P‐NCM523 and Al@EIC‐NCM523 simultaneously illustrate two types of fluorine, Polyvinylidene Fluoride (PVDF) at 687.1 eV and Li_x_PF_y_/Li_x_PF_y_O_z_ at 685 eV are detected in both samples after 300 cycles, which are the main hydrolysis products of the LiPF_6_ in the electrolyte (Figure 4e,f). Significantly, Al@EIC‐NCM523 exhibits lower Li_x_PF_y_/Li_x_PF_y_O_z_ peak intensity than P‐NCM523 (Figure 4e), which corresponds to fewer LiPF_6_ decomposition and confirms that Al@EIC surface construction effectively restrains the electrolyte decomposition and enhance the CEI stability. Another noteworthy finding is that a smaller peak area of Ni^2+^ is noticed for Al@EIC‐NCM523 compared to that of P‐NCM523 after cycling, which indicates that layered structure retention in the surface of Al@EIC‐NCM523 after cycling is higher than that of P‐NCM523, further proving that the surface crystal structure degradation is restricted by our proposed strategy (Figure 4g).

Lastly, the electrochemical impedance spectra (EIS) of P‐NCM523 and Al@EIC‐NCM523 before and after 200 cycles at 10 C are performed in the frequency range of 0.1 Hz to 100 000 Hz (Figure S17, Supporting Information). All the impedance spectra are fitted with the equivalent circuit to calculate the values of R_Ω_, R_s_, and R_ct_, which represent the resistance of the electrolyte solution, the resistance of the CEI film on the surface, and the charge transfer resistance, respectively (Table S7, Supporting Information). As a result, the R_s_ value of P‐NCM523 greatly increases from 9.364 Ω to 16.05 Ω after 200 cycles, which is mainly caused by interface deterioration after cycling. On the contrary, Al@EIC‐NCM523 displays substantially decreased values of R_s_ in both initial and cycled states, that is, from 9.161 Ω slightly increased to 11.56 Ω, demonstrating the improvement of interface stability and enhanced charge transport kinetics in Al@EIC‐NCM523 LIBs.^[^ ^52^ ^]^ All findings and analyses strongly show that our proposed strategy is a promising alternative for achieving both high‐voltage and high‐rate performance in NCM523, which involves the regulation of ion transport at the cathode interface through the utilization of ion‐electron dual‐conductive layer, along with the implementation of a crystalline structure stabilization strategy.

## 3 Conclusion

In this study, we harnessed ALD to intricately construct a layer of EIC coating on the surface of NCM523 cathode material, while simultaneously achieving a gradient Al doping in its bulk phase through meticulous thermal treatment. The EIC's dual‐conductive behavior and its underlying mechanism were rigorously elucidated via theoretical calculations, showing that EIC exhibited metal‐like electronic conductivity, along with accelerated Li^+^ ions migration kinetics at the interface between NCM523 and electrolyte. SEM and TEM results demonstrate the effective enhancement of interface stability and crystal structure of NCM523 by the Al@EIC layer. Moreover, in situ XRD and etching XPS technique reveal the mechanisms through which EIC facilitates ion transport at the interface, and the evolution of Al@EIC‐NCM523 crystal structure under elevated high‐voltage (4.6 V). As a result, an unprecedented rate performance of the as‐designed Al@EIC‐NCM523 at 4.6 V (114.7 mAh g^−1^ at 20 C), and remarkable cycling performance (capacity retention of 74.72% at 10 C after 800 cycles) are achieved. Furthermore, the practical application of Al@EIC‐NCM523||graphite pouch cells underscored its outstanding cyclic stability. This stability is attributed not only to the intrinsic structural stability of medium nickel ternary cathode but also to the enhanced electronic conductivity and facilitated Li^+^ ions diffusion due to EIC and Al doping. This approach promises to yield batteries that are not only competitively priced but also excel in high‐energy‐density and high‐power density, simultaneously addressing the critical demands of modern energy storage systems.

## Conflict of Interest

The authors declare no conflict of interest.

## Supporting information

Supporting Information

## Data Availability

The data that support the findings of this study are available from the corresponding author upon reasonable request.
