# Supplementary material for: Regulation of Interface Ion Transport by Electron Ionic Conductor Construction toward High‐Voltage and High‐Rate LiNi0.5Co0.2Mn0.3O2 Cathodes in Lithium Ion Battery
Source: Adv Sci (Weinh). 2024 Jun 5;11(30):2402380. doi: 10.1002/advs.202402380 (PMC11321621; doi:10.1002/advs.202402380)
Supplement: Supplementary file 1 — Supporting Information [file ADVS-11-2402380-s001.docx]

**Supplementary Information**

**Regulation of Interface Ion Transport by Electron Ionic Conductor Construction towards High-Voltage and High-Rate LiNi_0.5_Co_0.2_Mn_0.3_O_2_ Cathodes in Lithium Ion Battery**

Yunan Tian^a, b^, Yuyu Li^*, a, b^, Huasen Shen^a, b^, Xiangxin Cheng^a, b^, Yiming Cheng^a, b^, Wen Zhang^c^, Peng Yu^c^, Zehui Yang^d^, Lixing Xue^e^, Yameng Fan^f^, Lingfei Zhao^f^, Jian Peng*^, f, g^, Jiazhao Wang^f^, Zhaohuai Li^*, a, b^, Ming Xie*^, b^, Huakun Liu^f,h^ and Shixue Dou^f,h^

^a^State Key Laboratory of Precision Blasting, Jianghan University, Wuhan, 430056, China

^b^Hubei Provincial Engineering Research Center of Surface and Interface Regulation Technology and Equipment for Renewable Energy Materials, Jianghan University, Wuhan, 430056, China

^c^State Key Laboratory of Material Processing and Die & Mold Technology, School of Materials Science and Engineering, Huazhong University of Science and Technology, Wuhan, 430074 China

^d^Sustainable Energy Laboratory, Faculty of Materials Science and Chemistry, China University of Geosciences Wuhan, Wuhan, 430074, China.

^e^Cornex New Energy Co., Ltd. Wuhan, 432099, China

^f^Institute for Superconducting and Electronic Materials, Australian Institute for Innovative Materials, University of Wollongong, Innovation Campus, Squires Way, North Wollongong, NSW, 2522, Australia

^g^Department of Mechanical and Materials Engineering, Western University, London, ON, N6A 5B9, Canada

^h^Institute of Energy Materials Science, University of Shanghai for Science and Technology, Shanghai, 200093, People’s Republic of China

**Experimental section**

**Materials**

The NCM523 were used in this experiment as received from BattFlex(Wuhan) Technology Co., Ltd. According to provided parameters, the size of NCM523 particles was 2.3 μm, and the specific surface area of the NCM523 was 0.1 m^2^g^−1^, we marked it as P-NCM523. Trimethyl phosphate (TMPO) and trimethyl aluminum (TMA) from Aimouyuan (Nanjing) as precursors in a GM100 100-gram grade powder ALD equipment (Yunmao Technology Co., Ltd.). Super P (SP) was purchased from AkzoNobel corporation. Carbon nanotubes (CNTs) were acquired from M-Grade MWNT's, and the number is NTL-12112. Al foil and Cu foil were purchased from China Energy Lithium Co., Ltd. Separator used was Celgard 2400 PP film. The electrolyte was a high voltage electrolyte labelled SS-HNSS029. Anhydrous ethanol (> 99.8%), butadiene styrene rubber and carboxymethyl cellulose were purchased from Xin Shen Shi corporation (Wuhan). PVDF, N-Methyl pyrrolidone (NMP) and dimethyl carbonate (DMC) were purchased from Sigma-Aldrich without further purification.

**Preparation of Al@EIC-NCM523**

AlPO_4_ was deposited on P-NCM523 at 250°C. The source temperature of TMPO (99.999%) was 90°C, while TMA (99.9999%) was kept at room temperature (RT). Deposition was achieved by TMA (5s pulse) - purge (10s) - TMPO (5s pulse) - purge (10s). Nitrogen (99.999%) was used as a purge gas with a flow rate of 200 sccm. The above process was repeated to control the deposition thickness of AlPO_4_ by varying the number of cycles. After the experiment, the optimal thickness was obtained for four cycles, approximately 0.6 nm. Transfer the coated sample in a muffle furnace and calcined in air at 300°C for 4h, marked as Al@EIC-NCM523.

**Theoretical calculations**

The density functional theory (DFT) calculations were completed by Vienna Ab-initio Simulation Package ^[1]^ and envisioned by VESTA ^[2]^ software. Perdew-Burke-Ernzerhof exchange-correlation functional ^[3]^ and projector augmented-wave (PAW) method ^[4]^ with a kinetic energy cutoff of 500 eV were employed. The k-mesh produced by Gamma scheme has a density of 0.04*2π/Å. The energy convergence threshold of electronic self-convergence iteration is 10^−5^ eV, and the force convergence threshold for geometry optimization is 0.02 eV/Å. Climbing Image Nudged Elastic Band method ^[5]^ was used to simulate the kinetic migration of Li. The involved NCM523 structure with 240 atoms was built with reference to a previous work which identified preferable cation ordering pattern of NCM523. ^[6]^ In addition, some verified proper calculation parameters in the work were referenced. The semi-empirical PBE+U method (U values: 5.96, 5.00, 5.10 eV for Ni, Co, Mn atoms, respectively) were used for NCM523 to compensate for the inefficient evaluation of PBE on the strong interaction of d-electrons. The initial magnetic moment setting for an antiferromagnetic NCM523 was applied. The initial structure of AlPO_4_ (optimized lattice parameter: I-4 space group (number: 82), a=b=4.97, c=7.40, α=β=γ=90^o^) was downloaded from the open-source Materials Project database. The position of the bottom two layers of atoms in Al@EIC-NCM523 heterostructure was fixed in DFT calculations.

**Assembly of cells**

Half-cell assembly: The cathode material and SP were mixed in a mortar for 15 min, and then mixed with PVDF dissolved in NMP with a mass ratio of cathode material: SP: PVDF=80:10:10. The resulting slurry was coated on Al foil with a mass load of 5.2mg cm^-2^ and dried at 100°C under vacuum for 12h.The cathode electrode was cut into discs with a diameter of 12mm. NCM523||Li half-cells were assembled as coin cells (CR2032) in an argon-filled glove box.

Pouch-cell Assembly: The cathode electrode is made by mixing the active material (NCM523), conductive carbon (SP), CNTs and PVDF at a weight ratio of 96:1.5:0.5:2 in NMP to form a uniform slurry, and then coating on an Al foil substrate. After vacuum drying at 120°C for 12h, the electrode membrane was cut into 50×50 squares with an area density of approximately 16.7mg cm^−2^.The anode electrodes were fabricated by mixing graphite, SP, CNTS, butadiene styrene rubber and carboxymethyl cellulose in water at a weight ratio of 95:0.5:0.5:2.5:1.5 to form a homogeneous slurry. The slurry was then coated to a Cu foil substrate. After vacuum drying at 80°C for 12h, the electrode film was cut into 52×52 squares with an area density of about 10.0mg cm^−2^. The N/P ratio, representing the anode capacity to the NCM523 capacity, was approximately 1.17.

In-situ XRD: The in situ XRD experiment were identified using a PANalytical X-ray diffractometer and the patterns of in situ XRD patterns during first charging/discharging process was collected by using a specially designed cell with beryllium (Be) window, which was cycled between 3.0-4.6 V with a current density of 0.1 C (1C = 190 mAh g^−1^) and the 2θ scan range of 10-60°.

**Electrochemical tests**

For the half-cells, the galvanostatic charge-discharge tests were carried out using LANDCT2001A test system in a potential range of 3.0-4.6 V (versus Li/Li^+^) at 0.1C-20C at 25°C. Before long-term cycling at 5 C and 10C, the half-cells were cycled at 0.1 C (1C = 190 mAh g^−1^) for five times in the same voltage range. The pouch cells were tested in a voltage range of 3.0-4.5 V and were cycled at 0.1 C for five cycles before long-term cycling at 0.5 C. The CV and EIS measurements were tested using Autolab PGSTAT302N at 25°C. CV tests for calculating the diffusion coefficient of Li^+^ ion in different NCM523 samples were conducted with a scanning rate ranging from 0.1 to 1.6 mV s^−1^ at a voltage range of 3.0-4.6 V. Other CV curves were obtained with a scanning rate of 0.1 mV s^−1^ at a voltage range of 3.0-4.6 V. LSV tests were obtained with a scanning rate of 0.1 mV s^−1^ at a voltage range of 0-5.0 V. EIS tests were conducted at a frequency range of 0.1 Hz to 100 000 Hz.

**Materials characterizations**

SEM (Quattro S) was used to observe the surface morphology at an accelerating voltage of 15 kV. Its microstructure and elemental distribution were studied using 200kV TEM (tecnaiF20) and Energy Dispersive X-ray spectroscopy(EDS). The XRD measurements were conducted using BrukerD8Advance with Cu Kα radiation (λ = 1.5406 Å), in the 2θ range of 10-80°. Rietveld refinements of XRD results were obtained using the General Structure Analysis Software (GSAS) package with the EXPGUI interface. The surface chemical valence state was analyzed by XPS (Thermo Fisher Scientific K-Alpha) under Al-Ka radiation (1486.6eV), all calibrated with a C1s peak of 284.8eV. In addition, the Ar-ion etching was performed to obtain depth-dependent valence information and elemental distribution from samples. The resistivity is measured on a sample disc (0.1 g sample per disc under 5 MPa) by the four-point probe resistance tester, and the data obtained is the average of three measurements, the humidity is 65% RH. After 300 cycles, the cycled electrodes were removed from the batteries, rinsed several times with DMC solvent in an argon-filled glove box, and then dried for testing.

**Supplementary Figures**


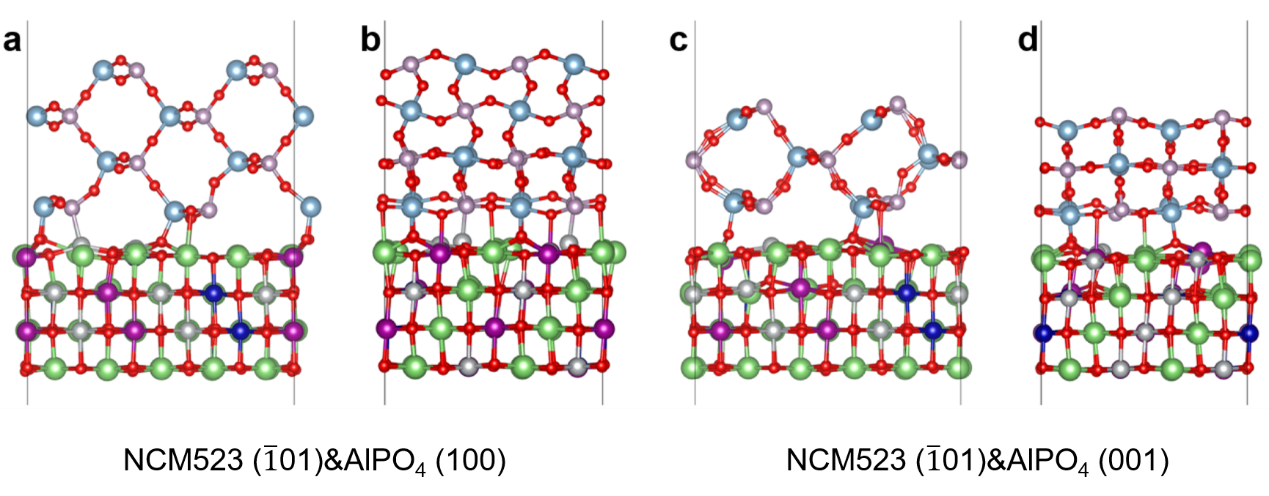


**Figure S1.** Optimized structures of NCM523/AlPO_4_ interface in different perspectives by matching NCM523 ($\bar{1}$01) lattice plane with (a, b) (100) and (c, d) (001) lattice planes of AlPO_4_, respectively.


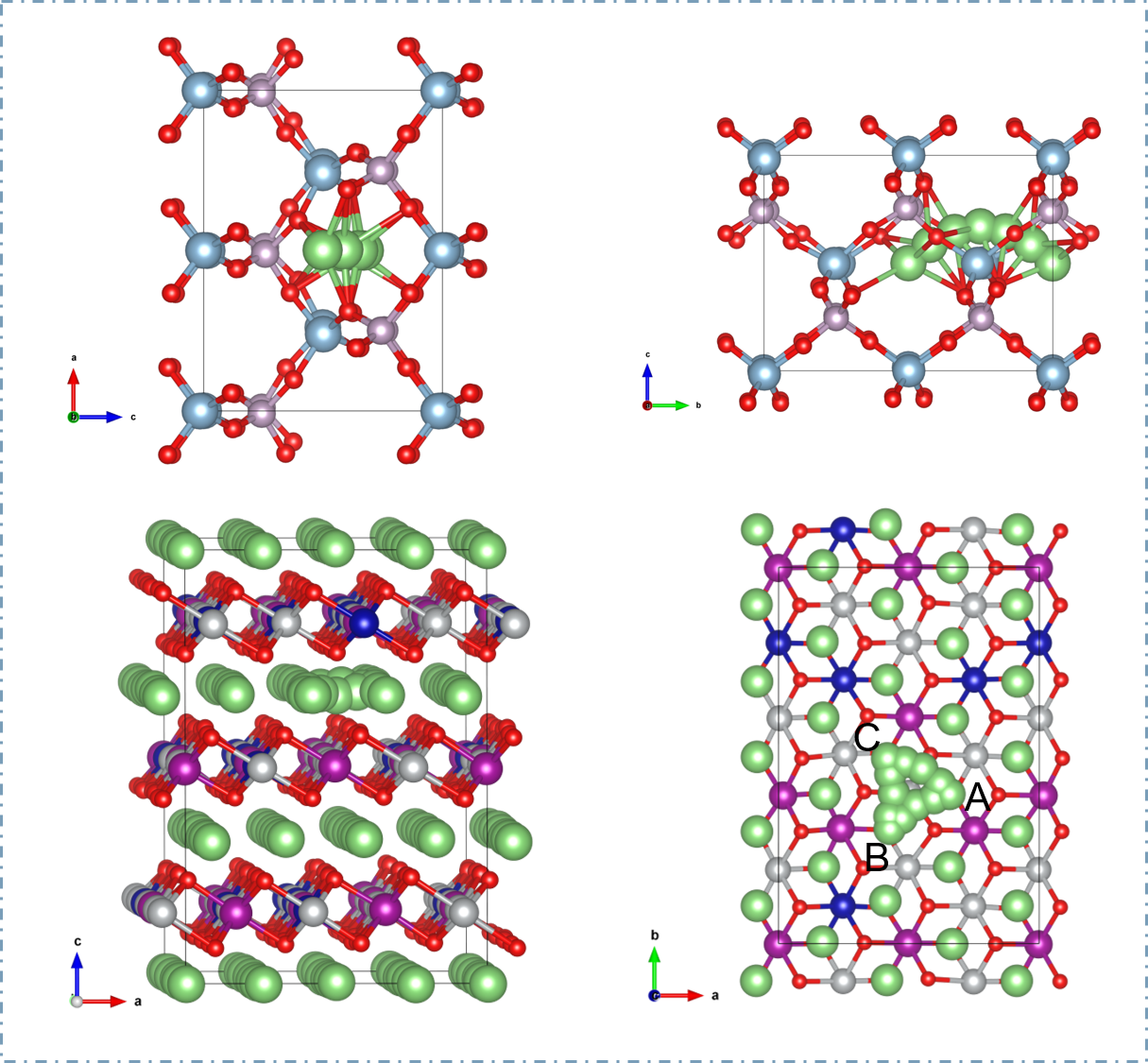


**Figure S2.** Li-ion diffusion pathways determined by DFT calculations. The initial structure of P-NCM523 is from ref, ^[6]^ and that of Al@EIC-NCM523 has similar ordering pattern to P-NCM523.


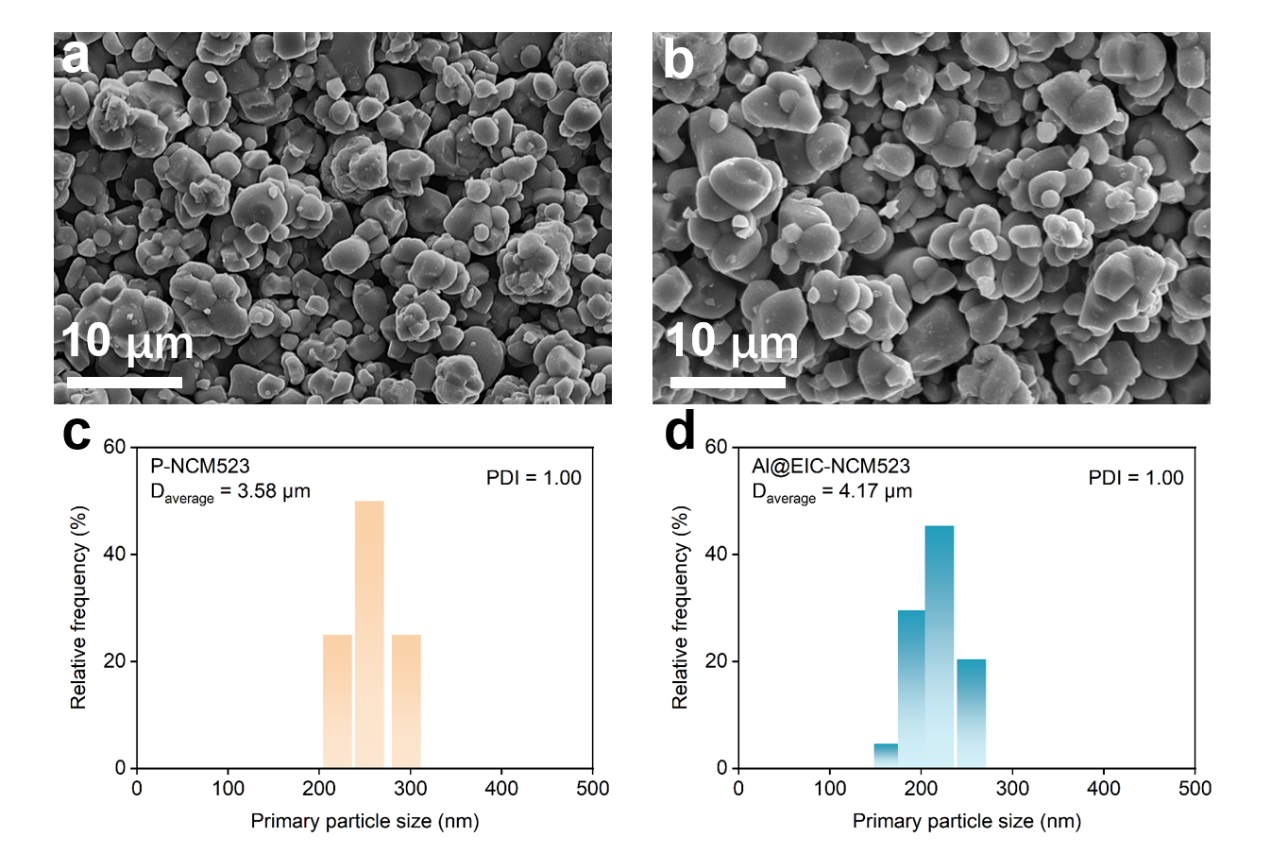


**Figure S3.** The SEM images of a) P-NCM523 and b) Al@EIC-NCM523 and the corresponding particle size distributions of c) P-NCM523 and d) Al@EIC-NCM523


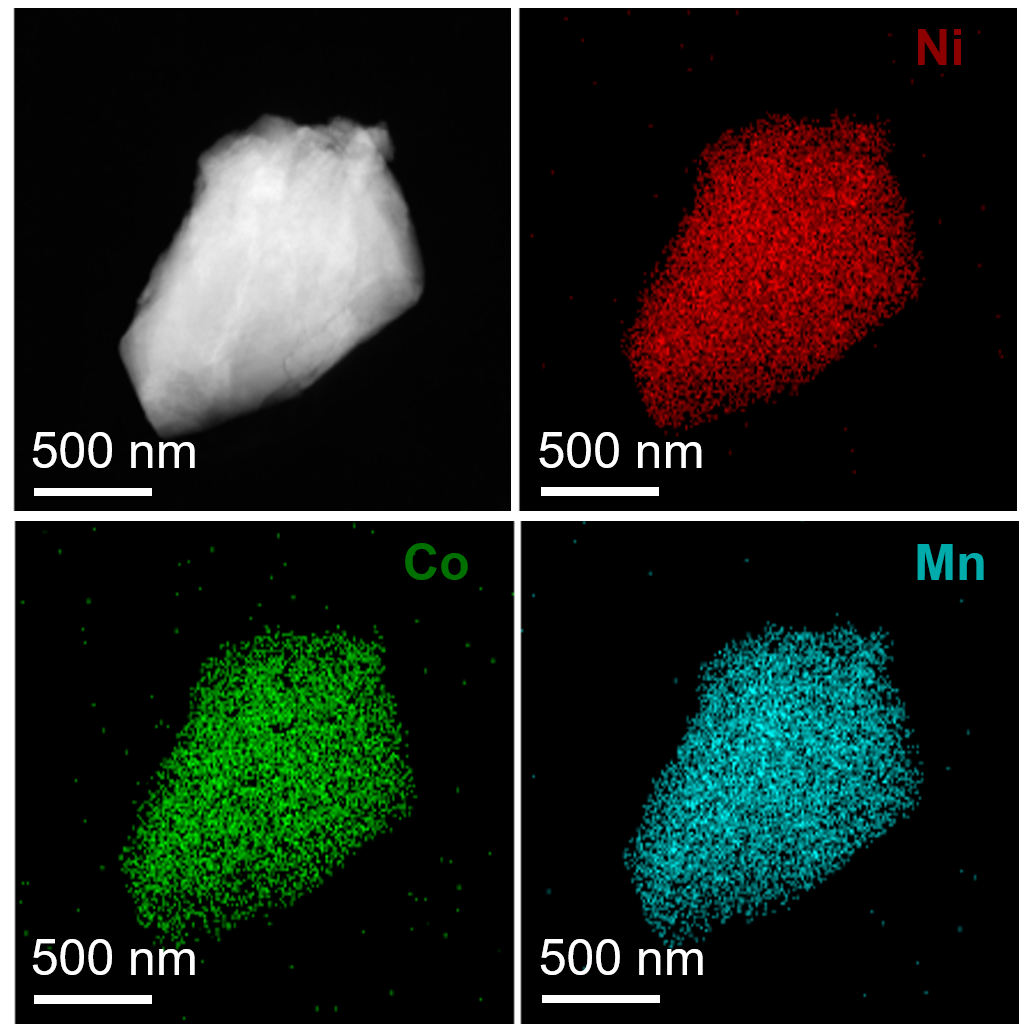


**Figure S4.** EDS mapping of P-NCM523.

**
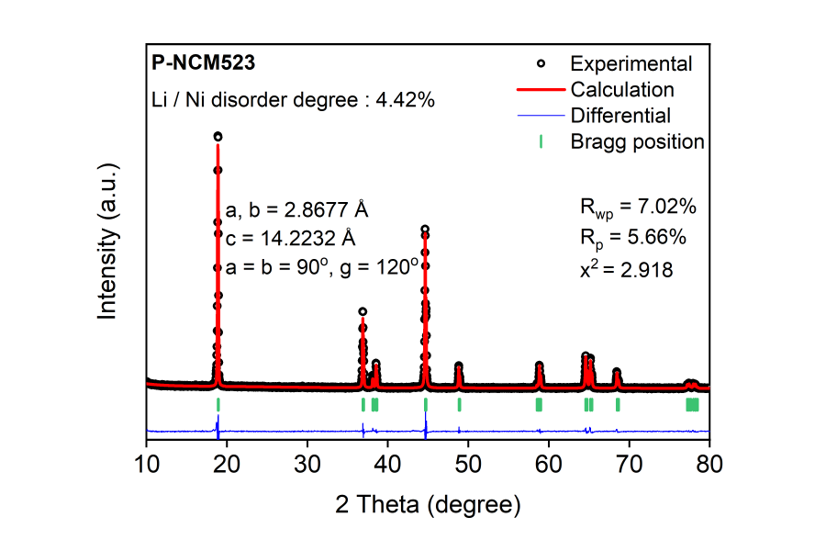
**

**Figure S5.** Rietveld refinement of the XRD patterns for P-NCM523.

**
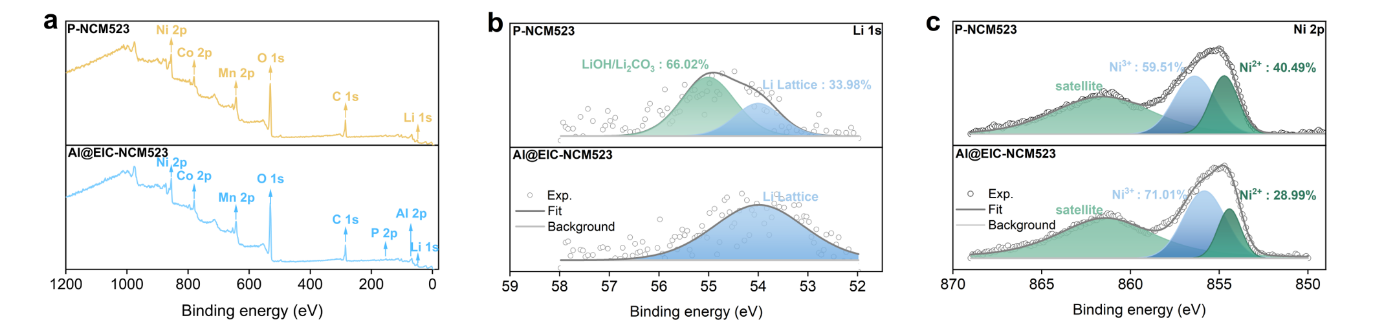
Figure S6.** (a) Original XPS survey spectra of P-NCM523 and Al@EIC-NCM523. (b) Li 1s and (c) Ni 2p XPS spectra of P-NCM523 and Al@EIC-NCM523.


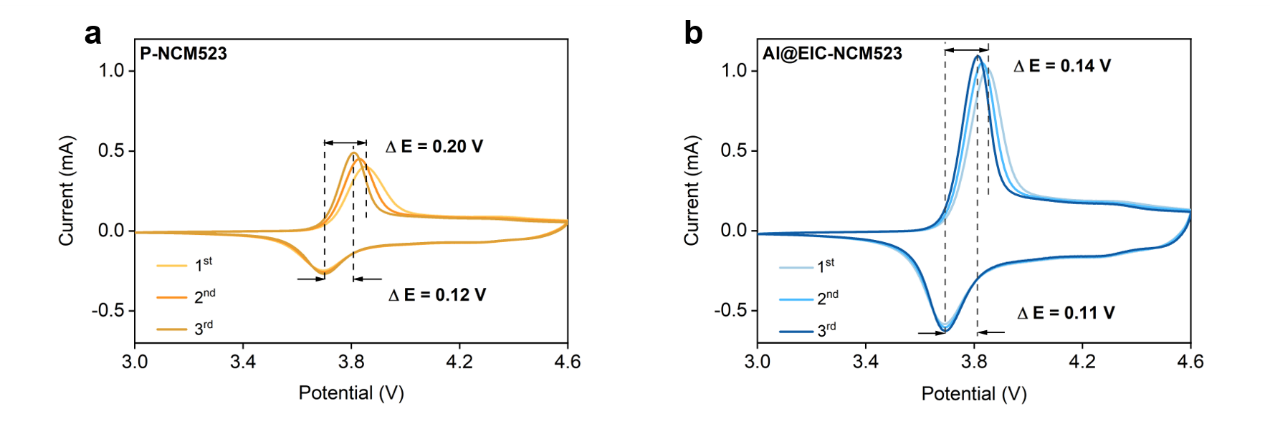


**Figure S7.** CV curves of (a) P-NCM523 and (b) Al@EIC-NCM523 at 0.1 mV s^-1^.

**
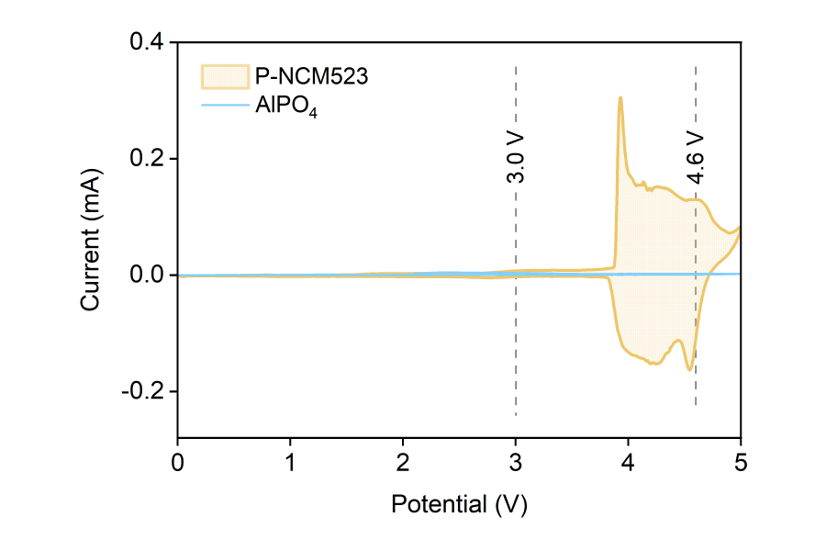
**

**Figure S8.** Line-scan voltammetry test of P-NCM523 and AlPO_4_ electrodes.

**
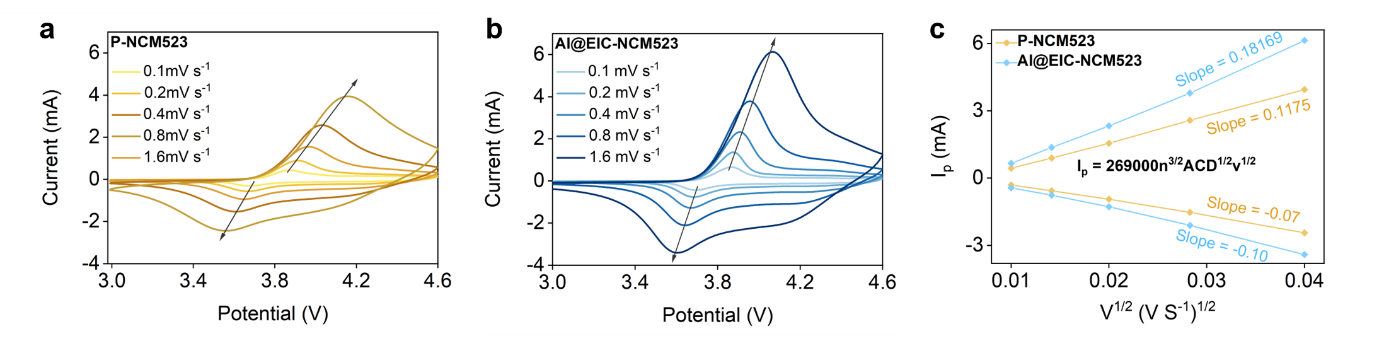
**

**Figure S9.** CV curves of (a) P-NCM523 and (b) Al@EIC-NCM523 at various scan rates. (c) The relationship of the peak current (I_p_) and the square root of scan rate (v^1/2^).


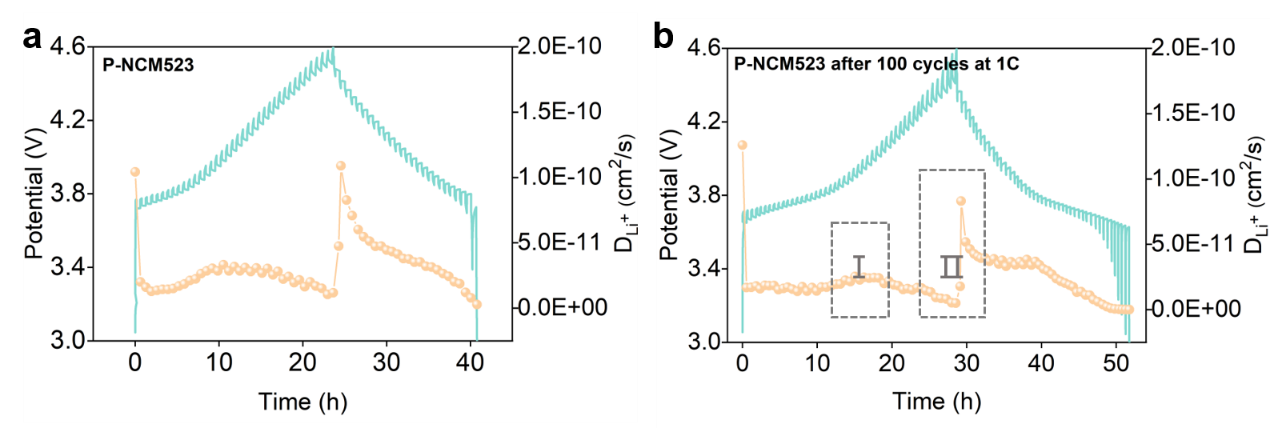


**Figure S10.** GITT measurements of P-NCM523 during the (a) initial and (b) after 100 cycles at 1C.


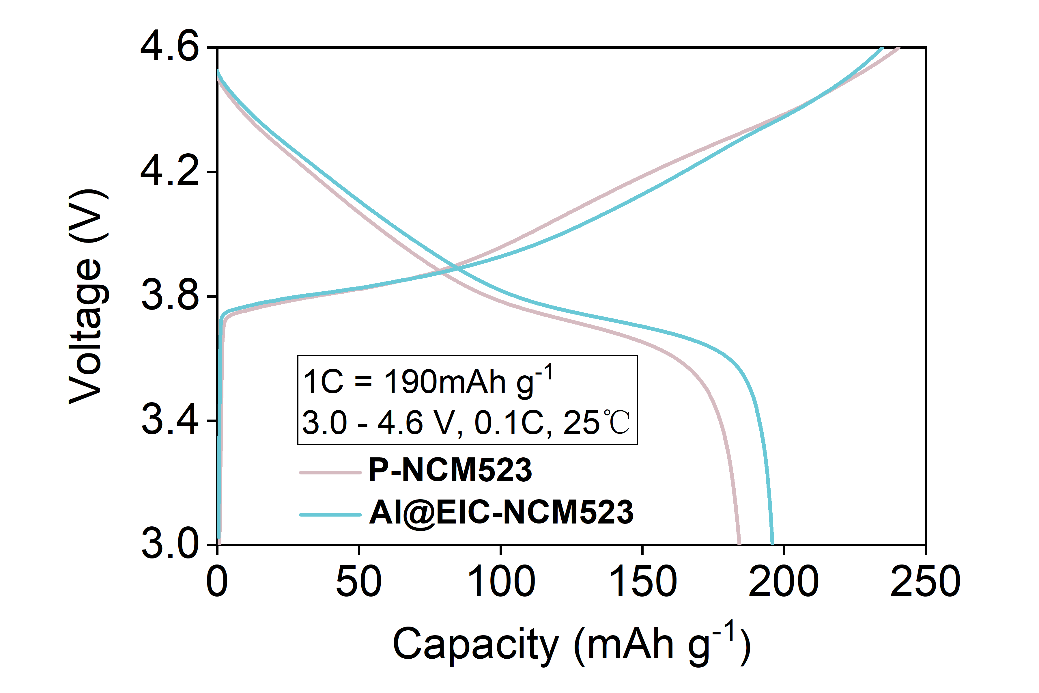


**Figure S11.** Selected charge–discharge curves of cycling tests between 3.0 – 4.6 V of P–NCM523 and Al@EIC-NCM523 at 0.1C.


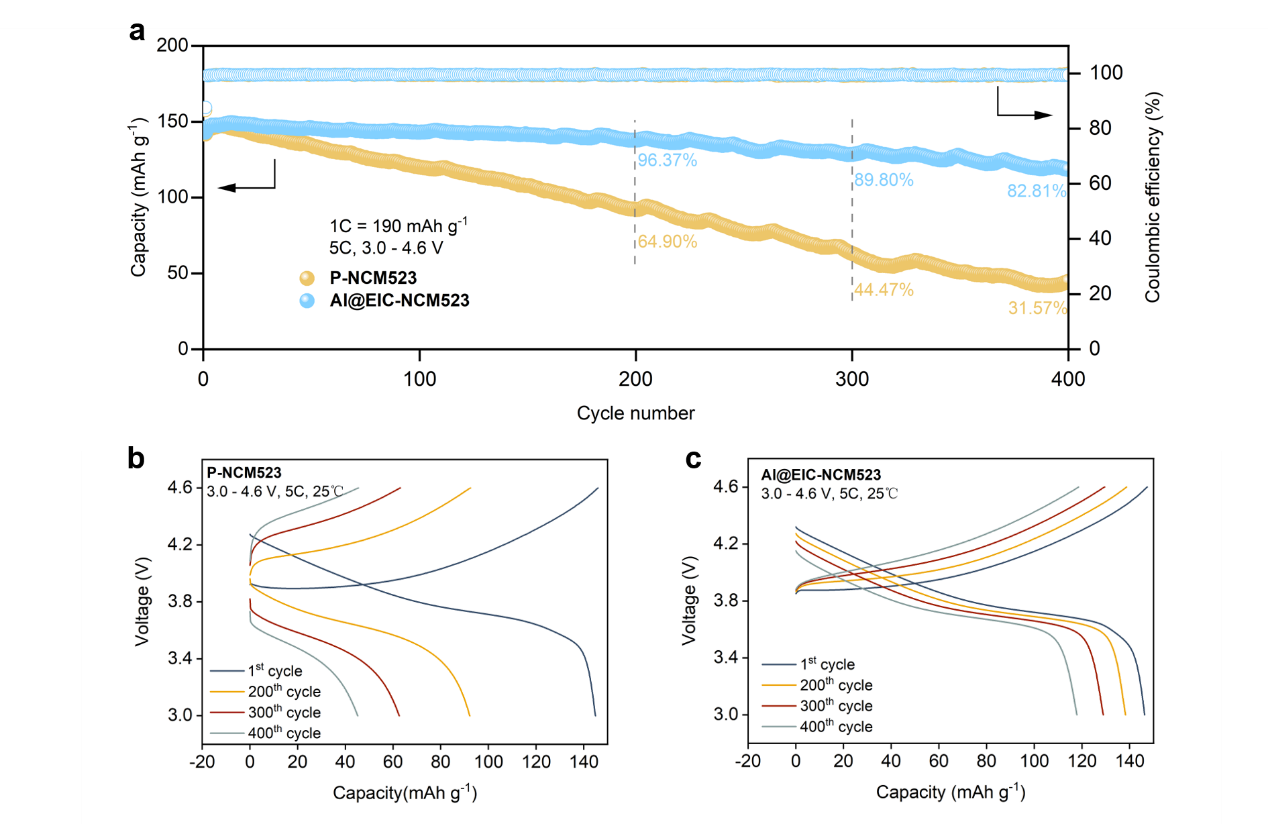


**Figure S12.** Comparison of (a) rapid charge and discharge performance at 5C and corresponding charge-discharge curves of the NCM || Li half-cells with (b) P-NCM523 and (c) Al@EIC-NCM523 between 3.0-4.6 V.


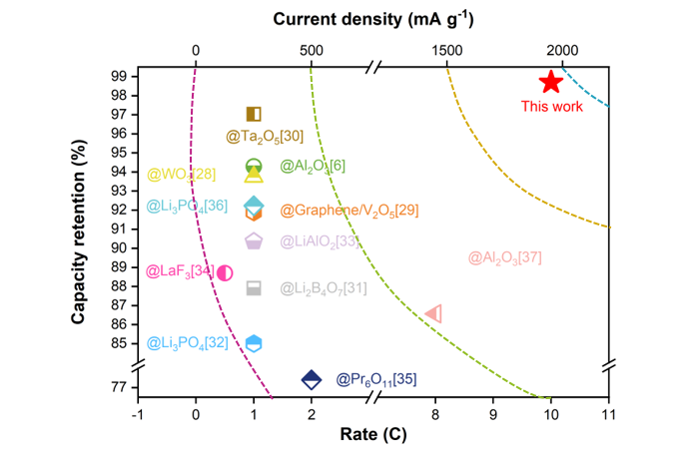


**Figure S13.** Comparison of the cycling performance of the Al@EIC-NCM523 with modified NCM523 in previous reports during 2020-2023. ^[7-17]^


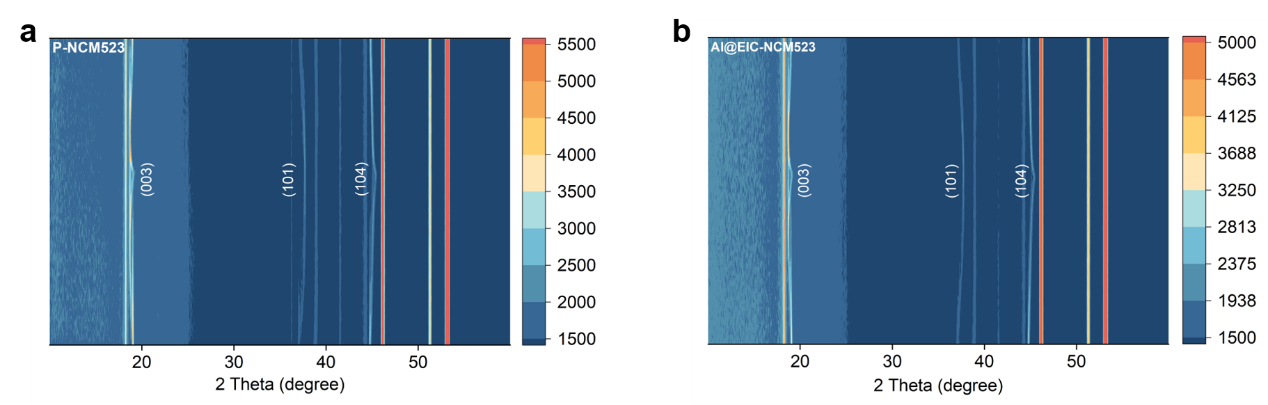


**Figure S14.** Contour plot for (a) P-NCM523 and (b) Al@EIC-NCM523 during the first charge and discharge process at 0.1 C between 3.0-4.6 V.


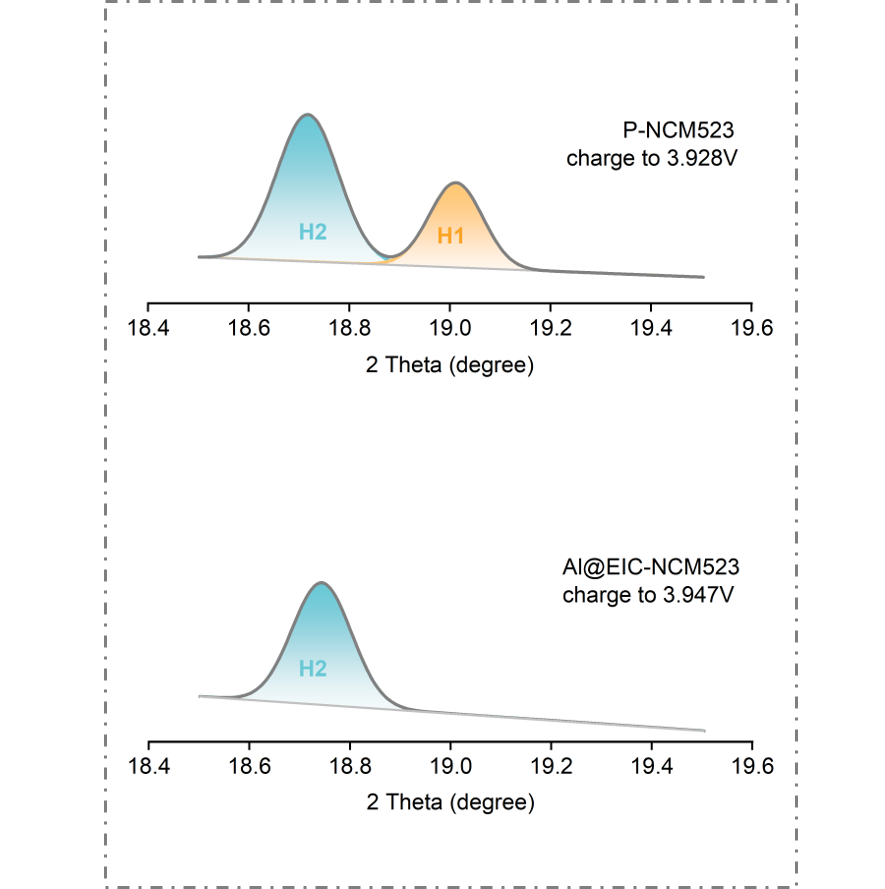


**Figure S15.** Overlays and deconvolutions of the (003) reflections peaks recorded by in situ XRD at H1-H2.


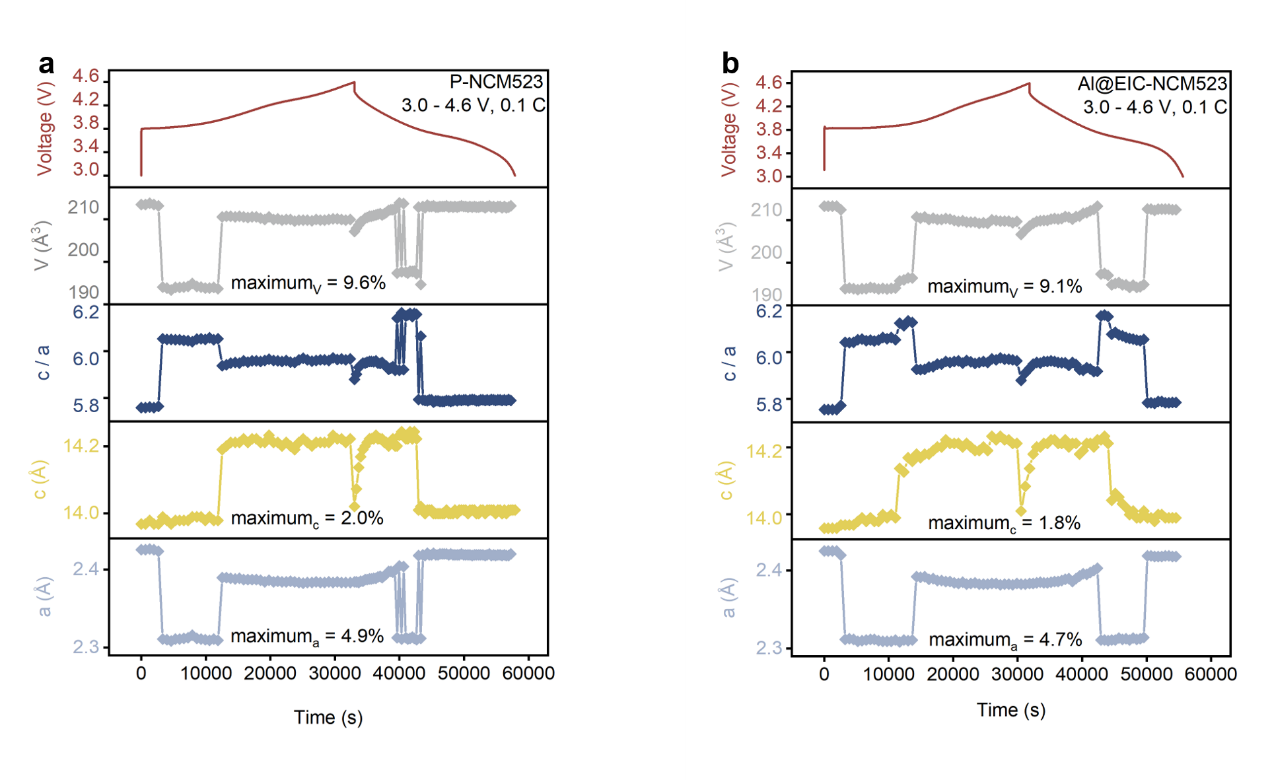


**Figure S16.** Calculated lattice parameters and volume change of (a) P-NCM523 and (b) Al@EIC-NCM523 samples during the charge and discharge process between 3.0-4.6 V.


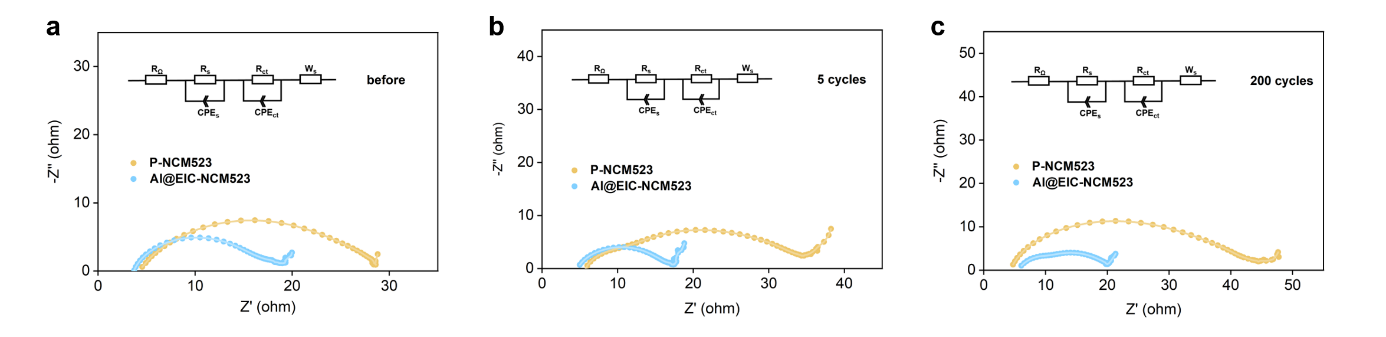


**Figure S17.** Plots of P-NCM523 and Al@EIC-NCM523 after the (a) 1st, (b) 5 th and (c) 200th cycles.

**Supplementary Tables**

**Table S1.** Rietveld refinement of P-NCM523 and Al@EIC-NCM523.

|  | a (Å) | b (Å) | c (Å) | c / a | I(003) / I(104) | Li / Ni disorder |
| --- | --- | --- | --- | --- | --- | --- |
| P-NCM523 | 2.8677 | 2.8677 | 14.2232 | 4.9598 | 1.5150 | 4.42% |
| Al@EIC-NCM523 | 2.8674 | 2.8674 | 14.2223 | 4.9600 | 1.7872 | 1.26% |

**Table S2.** Variation of height, conductivity and resistivity of P-NCM523 at different pressures.

| P-NCM523 | Height (mm) | Pressure (Mpa) | Resistivity (Ω cm^-1^) | Conductivity (μS cm^-1^) |
| --- | --- | --- | --- | --- |
| 1 | 0.56 | 1.98 | 87318 | 11.502 |
| 2 | 0.53 | 3.98 | 43558 | 23.208 |
| 3 | 0.51 | 5.99 | 28432 | 35.171 |
| 4 | 0.5 | 7.97 | 20869 | 47.923 |
| 5 | 0.48 | 9.99 | 16630 | 60.173 |
| 6 | 0.48 | 11.96 | 13425 | 74.55 |
| 7 | 0.47 | 13.97 | 11342 | 88.215 |
| 8 | 0.47 | 15.99 | 9429.8 | 106.04 |
| 9 | 0.46 | 17.99 | 8793.2 | 113.83 |
| 10 | 0.46 | 19.96 | 7759.8 | 129.05 |
| 11 | 0.45 | 21.95 | 7084.1 | 141.4 |
| 12 | 0.45 | 23.94 | 6393.2 | 156.72 |
| 13 | 0.44 | 25.93 | 5877.1 | 170.59 |
| 14 | 0.44 | 27.94 | 5367.5 | 186.82 |
| 15 | 0.43 | 29.93 | 5053.5 | 198.46 |

**Table S3.** Variation of height, conductivity and resistivity of Al@EIC-NCM523 at different pressures.

| Al@EIC-NCM523 | Height (mm) | Pressure (Mpa) | Resistivity (Ω cm^-1^) | Conductivity (μS cm^-1^) |
| --- | --- | --- | --- | --- |
| 1 | 0.54 | 2 | 20563 | 48.631 |
| 2 | 0.52 | 3.99 | 11339 | 88.214 |
| 3 | 0.5 | 6 | 8096.8 | 123.58 |
| 4 | 0.5 | 7.99 | 6193.7 | 161.64 |
| 5 | 0.49 | 9.97 | 5137.8 | 194.99 |
| 6 | 0.49 | 11.98 | 4328.7 | 231.6 |
| 7 | 0.49 | 13.96 | 3744.3 | 267.95 |
| 8 | 0.49 | 15.97 | 3421.9 | 292.86 |
| 9 | 0.48 | 17.97 | 3009 | 332.36 |
| 10 | 0.48 | 19.99 | 2721.5 | 367.47 |
| 11 | 0.48 | 21.98 | 2499.9 | 400.01 |
| 12 | 0.48 | 23.98 | 2280.1 | 438.59 |
| 13 | 0.48 | 25.9 | 2129.9 | 469.56 |
| 14 | 0.48 | 27.92 | 1970.9 | 507.43 |
| 15 | 0.48 | 29.93 | 1834.8 | 545.06 |

**Table S4.** Summary of the electrochemical performance of Al@EIC-NCM523 half-cell operating with a cut-off voltage higher than 4.5 V during 2020-2023. ^[7-17]^

| Modifying methods | Voltage  (V) | Current density  (mA g^-1^) | Cycle number | Capacity retention  (%) | Ref. |
| --- | --- | --- | --- | --- | --- |
| Al_2_O_3_ coating | 4.5 | 150 | 1000 | 94.29% | ^[13]^ |
| Li_3_PO_4_ coating | 4.6 | 170 | 200 | 85.00% | ^[15]^ |
| WO_3_ coating | 4.5 | 180 | 220 | 93.75% | ^[12]^ |
| Ta_2_O_5_ coating | 4.5 | 180 | 220 | 97.02% | ^[8]^ |
| Graphene + V_2_O_5_ coating | 4.5 | 180 | 205 | 91.89% | ^[9]^ |
| Al_2_O_3_ coating | 4.6 | 1240 | 220 | 86.57% | ^[16]^ |
| Li_2_B_4_O_7_ coating | 4.5 | 200 | 100 | 87.90% | ^[17]^ |
| Pr_6_O_11_ coating | 4.5 | 400 | 100 | 77.40% | ^[14]^ |
| Li_3_PO_4_ coating | 4.7 | 160 | 100 | 92.23% | ^[10]^ |
| LiAlO_2_ coating | 4.5 | 160 | 100 | 90.36% | ^[7]^ |
| LaF_3_ coating | 4.6 | 90 | 100 | 88.70% | ^[11]^ |
| This work | 4.6 | 1900 | 200 | 98.69% |  |

**Table S5.** Summary of the electrochemical performance of NCM half-cell operating with high rate. ^[18-24]^

| Materials | Rate  (C) | Voltage  (V) | Current density  (mA g^-1^) | Cycle number | Capacity retention (%) | Capacity  (mAh g^-1^) | Ref |
| --- | --- | --- | --- | --- | --- | --- | --- |
| NCM 622 | 10 | 4.5 | 2000 | 100 | 54.70 | 78.80 | ^[24]^ |
| NCM 523 | 10 | 4.6 | 1600 | 100 | 67.68 | 104.32 | ^[21]^ |
| NCM 523 | 10 15 | 4.3 | 1600 2388.06 | 150 | 89.30 77.83 | 98.50 76.90 | ^[23]^ |
| NCM 622 | 10 | 4.3 | 1800 | 120 | ~90.00 | ~108.00 | ^[22]^ |
| NCM 111 | \ | 3 | 1000 | 500 1000 | 75.00 56.00 | 56.25 42.00 | ^[18]^ |
| NCM 523 | 10 | 4.5 | 1600 | 200 | 50.70 | 68.45 | ^[19]^ |
| NCM 523 | 10 | 4.3 | 1600 | 500 | 79.04 | 95.62 | ^[20]^ |
| This work | 10 | 4.6 | 1900 | 500 800 | 89.19 74.72 | 130.13 109.02 |  |

**Table S6.** The parameters of NCM523||graphite pouch cell.

| **Item** | **Information** | **Properties** | **Value** | **Mass** |
| --- | --- | --- | --- | --- |
| Cathode | 96wt% LiNi_0.5_Co_0.2_Mn_0.3_O_2_  1.5 wt% SP  2.0 wt% PVDF  0.5 wt% CNTs | Active material  areal weight  Total area  First discharge capacity  Areal capacity | 16.7 mg cm^−2^  25 cm^2^  190 mAh g^−1^  3.17 mAh cm^−2^ | 434.9 mg |
| Al foil | 10 μm | Areal weight | 3.01 mg cm^−2^ | 75.33 mg  (contain tap) |
| Anode | 95wt% Graphite  0.5 wt% SP  0.5 wt% CNTs  2.5 wt%  butadiene styrene rubber  1.5 wt%  carboxymenthyl cellulose | Specific capacity  Areal capacity  N/P ratio | 372 mAh g^−1^  3.97 mAh cm^−2^  1.17 | 288.42 mg  (contain tap) |
| Cu foil | 6 μm | Areal weight | 6.64 mg cm^−2^ | 179.6 mg |
| Separator | 20 μm | Areal weight | 1.30 mg cm^−2^ | 35.1 mg |
| Electrolyte | Solvent (V/V/V):  30% EC+30% DEC+40% EMC  Lithium salt (1M):  100wt% LiPF6  Additive: 2wt% VC | E/C ratio | 3.00 g Ah^−1^ | 0.45 g |
|  |  |  | **Total mass** | 4.86 g |
| Cell | Cell level | Average voltage  Capacity  Cell energy | 3.70 V  **150 mAh**  **115.63 Wh kg^−1^** |  |
|  |  |  | **Total mass** | 3.36 g |
| Cell | Cell level | Average voltage  Capacity  Cell core energy | 3.70 V  **150 mAh**  **154.17 Wh kg^−1^** |  |

**Table S7.** Fitted values of R_Ω_, R_s_, and R_ct_ in P-NCM523 and Al@EIC-NCM523 half-cell at different cycles.

|  | R_Ω_(Ω) | R_s_(Ω) | R_ct_(Ω) | CPE_s_(µF) | CPE_ct_(µF) | W_s_(Ω/cm^2^) |
| --- | --- | --- | --- | --- | --- | --- |
| P-NCM523 | 4.779 | 9.364 | 13.89 | 0.91 | 0.78 | 1.34 |
| Al@EIC-NCM523 | 3.942 | 9.161 | 5.54 | 0.95 | 0.70 | 0.34 |
| 5 cycles P-NCM523 | 4.706 | 14.56 | 22.22 | 0.96 | 0.77 | 21.01 |
| 5 cycles Al@EIC-NCM523 | 5.106 | 5.017 | 6.55 | 1.01 | 0.75 | 2.70 |
| 200 cycles P-NCM523 | 5.398 | 16.05 | 30.31 | 0.66 | 0.57 | 51.02 |
| 200 cycles Al@EIC-NCM523 | 4.886 | 11.56 | 17.38 | 0.67 | 0.35 | 11.74 |

**References**

[1] G. Kresse, J. Furthmüller, *Phys. Rev. B* **1996**, 54, 11169.

[2] J. P. Perdew, K. Burke, M. Ernzerhof, *Phys. Rev. Lett.* **1996**, 77, 3865.

[3] P. E. Blöchl, *Phys. Rev. B* **1994**, 50, 17953.

[4] K. Momma, F. Izumi, *J Appl Crystallogr.* **2011**, 44, 1272.

[5] G. A. Henkelman, B. P. Uberuaga, H. J. J. o. C. P. Jónsson, *J. Chem. Phys.* **2000**, 113, 9901.

[6] M. Dixit, M. Kosa, O. S. Lavi, B. Markovsky, D. Aurbach, D. T. Major, *Phys. Chem. Chem. Phys.* **2016**, 18, 6799.

[7] H. Dong, H. Wang, J. Qi, J. Wang, W. Ji, J. Pan, X. Li, Y. Yin, S. Yang, *ACS Sustain. Chem. Eng.* **2022**, 10, 11587.

[8] Y. He, Y. Li, C. Xu, M. Zhu, W. Li, *Ceram. Int.* **2020**, 46, 14931.

[9] Y. Li, C. Xu, M. Dang, C. Yu, Y. He, W. Liu, H. Jin, W. Li, M. Zhu, J. Zhang, *Ceram. Int.* **2021**, 47, 21759.

[10] W. Qin, L. Feng, Z. Yang, J. Liu, Y. Liu, *Curr Appl Phys.* **2021**, 32, 36.

[11] H. Yang, H. Zhang, W. Zhao, *Ionics* **2023**, 29, 1335.

[12] Y. He, Y. Li, Y. Liu, N. Yao, J. Li, Y. Liu, *Appl. Surf. Sci.* **2020**, 508, 145259.

[13] L. Zhao, G. Chen, Y. Weng, T. Yan, L. Shi, Z. An, D. Zhang, *Chem. Eng. J.* **2020**, 401, 126138.

[14] G. Zhao, Y. Huang, W. Zhang, J. Gao, C. Xu, L. Shen, P. Lv, Y. Lin, Z. Huang, J. Li, *Chem. Eng. J.* **2022**, 441, 135929.

[15] H. Chen, L. Xiao, H. Chen, Y. Zhu, K. Xiang, H. Liao, *Electrochim. Acta* **2020**, 344, 136142.

[16] G. Cao, Z. Jin, J. Zhu, Y. Li, B. Xu, Y. Xiong, J. Yang, *J. Alloys Compd.* **2020**, 832, 153788.

[17] R. Deng, J. Tao, W. Zhong, L. Wen, Y. Yang, J. Li, Y. Lin, *J. Alloys Compd.* **2023**, 940, 168767.

[18] S.-F. Huang, Y. Lv, D. Tie, Y. Yu, Y.-F. Zhao, *Rare Metals* **2019**, 40, 65.

[19] B. Li, G. Li, D. Zhang, J. Fan, D. Chen, Y. Ge, F. Lin, C. Zheng, L. Li, *ChemistrySelect* **2019**, 4, 6354.

[20] L. Li, Z. Zhang, S. Fu, Z. Liu, *Appl. Surf. Sci.* **2019**, 476, 1061.

[21] L. Li, Z. Zhang, S. Fu, Z. Liu, Y. Liu, *J. Alloys Compd.* **2018**, 768, 582.

[22] Y. Liu, H. Lv, J. Mei, Y. Xia, J. Cheng, B. Wang, *J. Mater. Chem. A* **2023**, 11, 16815.

[23] L. Feng, Y. Liu, L. Wu, W. Qin, Z. Yang, J. Liu, *J. Alloys Compd.* **2021**, 881, 160626.

[24] Z. Cheng, F. Lv, N. Xu, Y. Liu, H. Xie, M. Wu, Y. Ma, Y. Zhang, L. Chen, *Appl. Surf. Sci.* **2020**, 524, 146556.
